# Synaptotagmin-1 attenuates myocardial programmed necrosis and ischemia/reperfusion injury through the mitochondrial pathway

**DOI:** 10.1038/s41419-025-07360-2

**Published:** 2025-01-26

**Authors:** Teng Sun, Jialei Li, Shuang Wang, Yu Han, Xiangyu Tao, Min Yuan, Zhijie Jing, Ting Liu, Yuehong Qi, Siqi Liu, Yanlin Feng, Jiasong Chang, Lan Zhou, Lijuan Gao, Jianyun Shi, Ruihong Ning, Jimin Cao

**Affiliations:** 1 Key Laboratory of Cellular Physiology at Shanxi Medical University, Ministry of Education, and the Department of Physiology, School of Basic Medicine, Shanxi Medical University, Taiyuan, China; 2 Laboratory Animal Center, Shanxi Medical University, Taiyuan, China; 3 First Hospital of Shanxi Medical University, Taiyuan, China; 4 The Anesthesiology Department of Shanxi Provincial People’s Hospital, Shanxi Medical University, Taiyuan, China; 5 Department of Cardiology, The Second Hospital of Shanxi Medical University, Taiyuan, China

**Keywords:** Necroptosis, Myocardial infarction, Mitochondria

## Abstract

Programmed necrosis/necroptosis greatly contributes to the pathogenesis of cardiac disorders including myocardial infarction, ischemia/reperfusion (I/R) injury and heart failure. However, the fundamental mechanism underlying myocardial necroptosis, especially the mitochondria-dependent death pathway, is poorly understood. Synaptotagmin-1 (Syt1), a Ca^2+^ sensor, is originally identified in nervous system and mediates synchronous neurotransmitter release. The later findings of Syt1 expressions in many non-neuronal tissues including muscles suggest that Syt1 may exert important functions beyond regulation of neurotransmitter release. Syt1 is highly expressed in cardiomyocytes and has been used as an extracellular molecular probe for SPECT imaging of cardiac cell death in acute myocardial infarction. However, whether Syt1 functions in the pathogenesis of cardiac disorders and what is the molecular etiology have not yet been clarified. We showed here that Syt1 expression was significantly down-regulated in mice I/R injured heart tissues, H_2_O_2_-challenged cardiomyocytes and hypoxia/reoxygenation (H/R)-damaged cardiomyocytes. Enforced expression of Syt1 significantly inhibited myocardial necrotic cell death and interstitial fibrosis, and improved cardiac function in mice subjected to I/R operation. In exploring the underlying mechanisms, we found that Syt1 interacted with Parkin and promoted Parkin-catalyzed CypD ubiquitination, thus inhibited mitochondrial membrane permeability transition pore (mPTP) opening and ultimately suppressed cardiomyocyte necrosis. We further found that Syt1 expression was negatively regulated by miR-193b-3p. MiR-193b-3p regulated cardiomyocyte necrosis and mPTP opening by targeting Syt1. Our present work revealed a novel regulatory model of myocardial necrosis composed of miR-193b-3p, Syt1, Parkin, and CypD, which may provide potential therapeutic targets and strategies for heart protection.

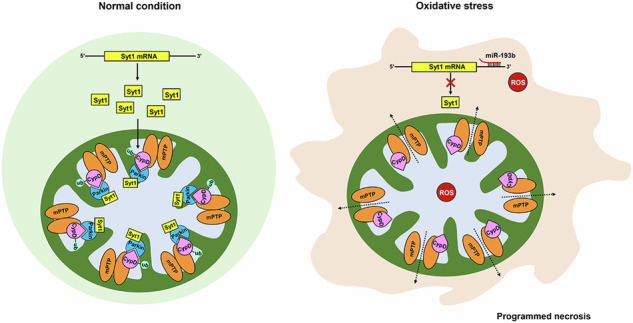

## Introduction

Cardiac cell death fundamentally contributes to the pathogenesis of heart diseases due to the limited proliferation capability of mature cardiomyocytes. Several morphologically distinct forms of cell death have been identified in damaged myocardium, including apoptosis, pyroptosis, necrosis and autophagy [1–3]. Necrosis is the prominent form of cell death in severely damaged hearts such as in ischemic hearts, ischemia/reperfusion injured hearts and diabetic hearts [1, 4, 5]. Necrosis has been considered as a passive, accidental and unregulated process for a long time. However, a growing body of evidences have demonstrated that necrosis could be tightly regulated, referred as programmed necrosis or necroptosis. Death receptor-dependent pathway has been identified as the canonical mechanism underlying necroptosis regulation [4, 5]. Recent advances have revealed that mitochondrial network is deeply involved in the regulation of programmed necrosis. Mitochondrial permeability transition pore (mPTP), a supramolecular channel spanning the inner and outer mitochondrial membranes, controls the solutes exchange between the mitochondrial matrix (MA) and the surrounding cytoplasm. However, the abnormally prolonged opening of mPTP results in loss of mitochondrial membrane potential, energetic deficits, organelle swelling, and eventually necrotic cell death [6, 7]. Reactive oxygen species (ROS) are the byproducts of normal mitochondrial metabolism and they are transported to cytoplasm through transient opening of mPTP. Excess ROS produced in infarcted and I/R injured hearts triggers mPTP prolonged opening, followed by necroptosis and impaired cardiac function [8, 9].

Cyclophilin-D (CypD) protein, encoded by the peptidyl-prolyl cis-trans isomerase F gene (*PPIF*), is a core functional component of mPTP complex and regulates mPTP opening. Increased or phosphorylated CypD obligates both cardiomyocytes and endothelial cells to undergo necrotic cell death by augmenting mPTP opening in myocardial ischemia/reperfusion injury [10, 11]. Mice deficient with CypD exhibit reduced loss of mitochondrial membrane potential, ameliorated mitochondria swollen, and improved cardiac function [12]. Deacetylation inactivation of CypD shuts down mPTP and protects hearts against aging and cardiac hypertrophy [13]. mPTP opening is suppressed upon disruption of interaction between CypD and mPTP, which inhibits mitochondrial ROS output and fibroblast activation in non-alcoholic steatohepatitis [14]. Our previous work has revealed that CypD-mPTP-dependent necrosis contributes to myocardial I/R injury and oxidative stress injury. In addition, mitochondrial-localized Parkin interacts with CypD and catalyzes its ubiquitination, preventing CypD from triggering mPTP opening. By targeting the CypD-mPTP signaling pathway, Parkin inhibits necrotic cell death and plays a cardiac protective role under oxidative stress [15]. However, the fundamental mechanisms underlying myocardial programmed necrosis, especially the mitochondrial pathway, needs to be further studied.

Synaptotagmin-1 (Syt1), a Ca^2+^ sensor with 2 tandemly arranged Ca^2+^-binding C2 domains (C2A and C2B), mediates synchronous neurotransmitter release [16, 17]. Originally, Syt1 has been demonstrated to regulate endocytosis [18], vesicle trafficking [19], membrane repair [20], and synaptic plasticity [21] during synaptic transmission. Syt1 and other members of the synaptotagmin family oligomerize into ring-like vesicle clamps to facilitate vesicle docking and inhibit membrane fusion [22, 23]. Syt1 is widely and deeply implicated in the pathogenesis of neurodevelopmental disorders [24–26] and alcohol addiction [27]. Recent advances have revealed that Syt1 probably participated in the regulation of oxidative stress. The oxidative stress imbalance in glioma was accompanied by down-regulation of Syt1 mRNA [28]. Syt1-positive neurons exhibit more axonal varices which is a key feature of neuronal susceptibility to oxidative stress [29]. Both the mRNA and the protein levels of Syt1 were decreased during oxidative stress induced by advanced glycation end-products (AGEs) in diabetic encephalopathy [30]. Bioinformatics analysis suggests a strong correlation between Syt1 and oxidative stress [28]. As early as 2006, Syt1 was identified to express in atrial cardiomyocytes from neonatal and adult rats [27]. In response to myocardial infarction, necrotic cardiomyocytes undergo cytoplasmic membrane rupture and anionic phospholipids exposure. Syt1 recognizes necrotic cells by its C2A domain binding to exposed anionic phospholipids. This property of Syt1 was used to synthesize an extracellular molecular probe for SPECT imaging of cardiac cell death in acute myocardial infarction [31]. However, the potential role of Syt1 in the pathogenesis of cardiac disorders and the molecular mechanism have not been clarified.

The present study aimed to investigate the role of Syt1 in cardiomyocyte programmed necrosis and the underlying mechanisms. Our data showed that Syt1-regulated mPTP opening, necrotic cell death and myocardial injury. In exploring the mechanisms, we found that Syt1 interacted with Parkin and participated Parkin-mediated CypD ubiquitination, which contributed to the anti-necrosis function of Syt1. We also identified miR-193b-3p as a regulator of Syt1 expression. Taken together, we revealed a novel regulatory model of cardiomyocyte programmed necrosis.

## Material and methods

### Mouse model of myocardial infarction and determination of infarct size

All animal experiments were performed using protocols that adhered to the standards of the National Institute of Health Guide for the Care and Use of Laboratory Animals, and the protocol was approved by the Animal Ethics Committee of Shanxi Medical University (SYDL2023014). Mice were housed under pathogen-free conditions, keeping the temperature in the breeding room at 21–26 °C, the humidity at 50%–60%, and providing a 12-h light and 12-h dark cycle. Seven-week-old male C57BL/6J mice were anesthetized with 2% isoflurane and infected with adenovirus expressing Syt1 or miR-193b-3p antagomirs. To perform adenovirus infection, after cross-clamping the aorta and pulmonary artery, adenoviruses (2 × 10^10^ moi) were injected into the aortic root using a catheter from the apex of the left ventricle. Hearts pumped in the closed system for 20 s. Five days later, mice were anesthetized again and subjected to ligation of the left anterior descending coronary artery (LAD) for 45 min, followed by 1 h of reperfusion. Then echocardiography and histological staining were performed. During the experiment, the mice were randomly grouped in accordance with the experimental design. The investigator was blinded to the group allocation of the mice. The sample size is described in the corresponding figure legend. No animals were excluded from the analysis.

### Construction of adenovirus

The coding sequence of S*yt1* mRNA was synthesized and cloned into ADV4(CMV/IRES-GFP) vector. The sense sequence of *Syt1* siRNA was 5′-GCCTTAATAGCCATAGCCATA-3′, and the antisense sequence was 5′-TATGGCTATGGCTATTAAGGC-3′. The sense sequence of scrambled S*yt1* siRNA was 5′-ACTACCGTTGTTATAGGTG-3′, and the antisense sequence was 5′-CACCTATAACAACGGTAGT-3′. The siRNAs or the scramble siRNAs were cloned into the ADV3 (U6/CMV-GFP&PURO) vector. HEK293 cells was used to produce the adenoviruses expressing *Syt1* or *Syt1* siRNAs.

### Echocardiography and histological staining

Echocardiography was carried out as we described previously [32]. In brief, 1–2 weeks after I/R injury, transthoracic echocardiography was performed under 2% isoflurane anesthesia to analyze cardiac structure and function using Prospect High Resolution Imaging System equipped with a 40 MHz probe. LV systolic internal diameter (LVIDs) and LV diastolic internal diameter (LVIDd) were measured. Fractional shortening (FS) of the LV was formulated as [(LVIDd−LVIDs)/LVIDd] × 100. Then mice were euthanized with an anesthetic overdose of isoflurane (5%) followed by cervical dislocation, and hearts were harvested and embedded in paraffin and sectioned at 6 μm thickness for histological staining according to the manufacturer’s instructions. Hematoxyline and eosin (H&E) (Solarbio, Beijing, China) staining and Masson trichrome staining (Solarbio, Beijing, China) were performed to assess interstitial fibrosis. The cross-sectional area of cardiomyocytes was detected using TRITC-conjugated wheat germ agglutinin (WGA) (Thermo Fisher, Massachusetts, America) staining. Evens blue/TTC staining was performed to analyze the infarct area 24 h after I/R. The re-ligature around the left coronary artery (LAD) was performed in all groups including experimental group, negative control (NC) group and sham group, and then hearts were injected with 2% evens blue from jugular vein. After rapidly frozen at −80 °C for 30 min, the tissue was sectioned at 2 mm thickness and incubated with 1% TTC at 37 °C for 15 min, followed by fixed in 10% neutral formaldehyde. Images were analyzed by ImageJ.

### PI staining of heart tissue and cardiomyocytes

Propidium iodide (PI) staining was performed to analyze necrotic cell death. Hearts were injected with PI (10 mg/kg) 1 h before I/R surgery termination to label the necrotic cells. After I/R, mice were euthanized with an anesthetic overdose of isoflurane (5%) followed by cervical dislocation, and the hearts were rapidly explanted, snap-frozen in liquid nitrogen, and sectioned at 6 μm thickness. α-actinin was used to stain cardiomyocytes and DAPI was used to identify nuclei. Magnification of 400× images were taken using a laser scanning confocal microscope (Olympus, Tokyo, Japan), and 100–200 cardiomyocytes from 30 to 50 fields were quantified by an investigator blinded to the sample. H9c2 cells were incubated with 1.5 μM PI for 20 min on ice in a dark chamber. After fixed in 4% paraformaldehyde, the sections were mounted with fluorescent mounting medium with DAPI. Images were taken using a laser scanning confocal microscope (Olympus, Tokyo, Japan).

### Detection of lactate dehydrogenase (LDH) activity

LDH activity were measured in serum of mice and supernatant of cultured cardiomyocytes using spectrophotometric kit (Nanjing Jiancheng, Jiangsu, China) according to the manufacturer’s instructions. In brief, 20 μL serum or 20 μL supernatant was prepared and successively incubated with the following reagents: 25 μL MA buffer and 5 μL coenzyme I at 37 °C for 15 min, 25 μL 2,4-dinitrophenylhydrazine at 37 °C for 15 min, and 250 μL 0.4 M NaOH at room temperature for 5 min. The LDH activity was assessed from the absorbance value at 450 nm.

### Cell culture and treatment

Embryonic cardiomyocyte-derived H9c2 cell line and HEK293 cells was cultured in Dulbecco’s modified Eagle’s medium (DMEM) (Hyclone, State of Utah, America) supplemented with 10% fetal bovine serum, 100 U/mL penicillin, 0.1 mg/mL streptomycin, and 110 mg/mL sodium pyruvate in a humidified atmosphere containing 5% CO_2_ at 37 °C. H9c2 cells were treated with 100 μM or 500 μM H_2_O_2_ for 12 h except as otherwise indicated. H9c2 cells were exposed to DMEM/F12 medium (without Glucose) for 12 h and then cultured in a hypoxic environment at 37 °C. Next, the cells were replaced with DMEM/F12 medium containing 5% fetal bovine serum, and cultured in a reoxygenated environment with 5% CO_2_ and 95% O_2_ at 37 °C for 6 h.

### Quantitative reverse transcription-PCR (qRT-PCR)

The levels of mature miRNAs were detected using Stem-loop qRT-PCR carried out on Applied Biosystems ABI Prism QuantStudio 3. Total RNA was extracted by Trizol reagent (Invitrogen, State of California, America). After DNase I digestion, RNAs were reverse transcribed into cDNA using PrimeScript^™^ RT reagent Kit (Takara, Kyoto, Japan) and specific primers. The RT primers of rat miR-193b-3p were 5′- GTCGTATCCAGTGCAGGGTCCGAGGTATTCGCACTGGATACGACGGGACT-3′. The RT primers of mouse miR-193b-3p were 5′-GTCGTATCCAGTGCAGGGTCCGAGGTATTCGCACTGGATACGACAGCGGG-3′. The levels of mature miR-193b-3p were detected by real-time PCR using TB Green^®^ *Premix Ex Taq*^™^ II (Takara). The primers of rat miR-193b-3p were: forward, 5′-AACTGGCCCACAAAGTCCC-3′; reverse, 5′-GTGCAGGGTCCGAGGT-3′. The primers of mouse miR-193b-3p were: forward, 5′-AACTGGCCCACAAAGTCCCGCT-3′; reverse, 5′-GTGCAGGGTCCGAGGT-3′. The data were normalized to that of U6. The RT primers of U6 were 5′-AACGCTTCACGAATTTGCGT-3′. The real-time PCR primer of U6 were: forward, 5′-GCTTCGGCAGCACATATACTAA-3′; reverse, 5′-AACGCTTCACGAATTTGCGT-3′.

### Detection of mitochondrial membrane potential (△Ψm)

JC-1 (Beyotime, Beijing, China) was used to detect the mitochondrial membrane potential (Δψm) according to the manufacturer’s instructions. Briefly, cells were exposed to JC-1 staining solution for 20 min. After washed twice with ice-cold JC-1 staining buffer, images were taken using a fluorescence microscope (Nikon, Kyoto, Japan) and analyzed by ImageJ software.

### Calcein-AM staining

The Calcein-AM/CoCl_2_ quenching technique (Beyotime) was used to detect the mPTP opening according to the manufacturer’s instructions. Briefly, the cells were incubated with 0.5× calcein-AM (calcein acetoxymethyl ester) and 1× CoCl_2_ (a cytosolic calcein fluorescence quencher) for 30 min at 37 °C, and then incubated with DMEM for 30 min at 37 °C. Images were taken using a fluorescence microscope (Nikon, Japan) and analyzed by ImageJ software.

### Mitochondrial swelling assay

A calcium-induced mitochondrial swelling assay was used to measure mPTP opening. The mitochondria were isolated using a mitochondria extraction kit (Solarbio, SM0020) according to the manufacturer’s instructions. The isolated mitochondria were resuspended in a swelling buffer containing 100 mM KCl, 50 mM MOPS, 5 mM KH_2_PO_4_, 0.5 µM EGTA, 1 mM MgCl_2_, 5 mM glutamate, and 5 mM malate, and then CaCl_2_ (200 µM) was added to the mitochondrial suspension. The absorbance at 520 nm was measured every 1 min for a total of 30 min.

### Western blotting

Cells were lysed in RIPA lysis buffer supplemented with 0.1 mM PMSF and a protease inhibitor cocktail (Abcam, Boston, America). Then proteins were undergone the following steps: quantification, SDS-PAGE electrophoresis and transferred to PVDF membranes. Blots were probed using the primary antibodies including anti-synaptotagmin-1 antibody (Abcam, Boston, America), anti-Parkin antibody (Abcam, Boston, America), anti-CypD antibody (Cell Signaling Technology, Massachusetts, America), anti-HA antibody (Cell Signaling Technology, Massachusetts, America), anti-actin antibody (Boster, Wuhan, China), anti-COX IV antibody (Beyotime, Beijing, China). The protein-antibody complexes were visualized using ECL western blotting substrate (Boster, Wuhan, China) on a Bio-Rad ChemiDoc system. The blotting bands were analyzed by the ImageJ software.

### Immunoprecipitation

Immunoprecipitation (IP) assay was performed using Pierce^®^ Classic IP Kit (Thermo Fisher Scientific, Massachusetts, America) according to the manufacturer’s instructions. In brief, a mixture of 2 μg antibody and 20 μL Protein A/G Plus Agarose was prepared and rotated at room temperature for 60 min. Cells were lysed in ice-cold IP Lysis/Wash buffer for 5 min. After centrifugation, 50 μL proteins were aliquoted for input, and the remaining proteins were incubated with antibody-crosslinked resin at 4 °C overnight. The IP products were eluted and subjected to western blotting. The antibodies used for immunoprecipitation were anti-synaptotagmin-1 antibody (Synaptic Systems, Goettingen, Germany), anti-Parkin antibody (Abcam, Boston, America), anti-CypD antibody (Cell Signaling Technology, Massachusetts, America), and anti-actin antibody (Bioss, Beijing, China). H9c2 cells were transfected with HA-tagged ubiquitin expressing vector, followed by infected with adenoviruses expressing Syt1 siRNAs or Parkin. Then immunoprecipitation was performed. The anti-HA antibody (Cell Signaling Technology, Massachusetts, America) was used to detect the HA -ubiquitin levels in CypD protein.

### Immunofluorescent staining

Cultured cardiomyocytes were incubated with 20 nM MitoTracker Red CMXRos (YEASEN, Shanghai, China) for 15 min at 37 °C and then fixed in 4% paraformaldehyde. After blocked with 5% BSA, cells were treated with primary antibodies including anti-Syt1 antibody (Abcam; Boston, America) and anti-Parkin antibody (Abcam, Boston, America) at 4 °C overnight. Subsequently, the cellular sample were exposed to the fluorescein isothiocyanate-conjugated secondary antibody and mounted with fluorescent mounting medium with DAPI. Images were taken using a laser scanning confocal microscope (Olympus, Kyoto, Japan).

### Dual-luciferase assay

The 3′UTR or the mutated 3′UTR of Syt1 mRNA were synthesized and cloned into psiCHECK-2 vector (Promega, Beijing, China) downstream the firefly luciferase gene. The vectors were transfected into HEK293 cells or cardiomyocytes using Lipofectamine 3000 (Thermo Fisher Scientific, Massachusetts, America). The chemiluminescence signal was measured using Dual-Glo^®^ Luciferase Assay System (Promega, Beijing, China) according to the manufacturer’s instructions.

### Statistical analysis

Data were expressed as mean ± standard deviation (SD) of at least five independent experiments. Student’s *t*-test was used for comparison of two groups. One-way ANOVA was used for multiple group comparisons. A value of *p* < 0.05 was considered statistically significant.

## Results

### Synaptotagmin-1 protects against myocardial programmed necrosis and I/R injury

**Fig. 1:**
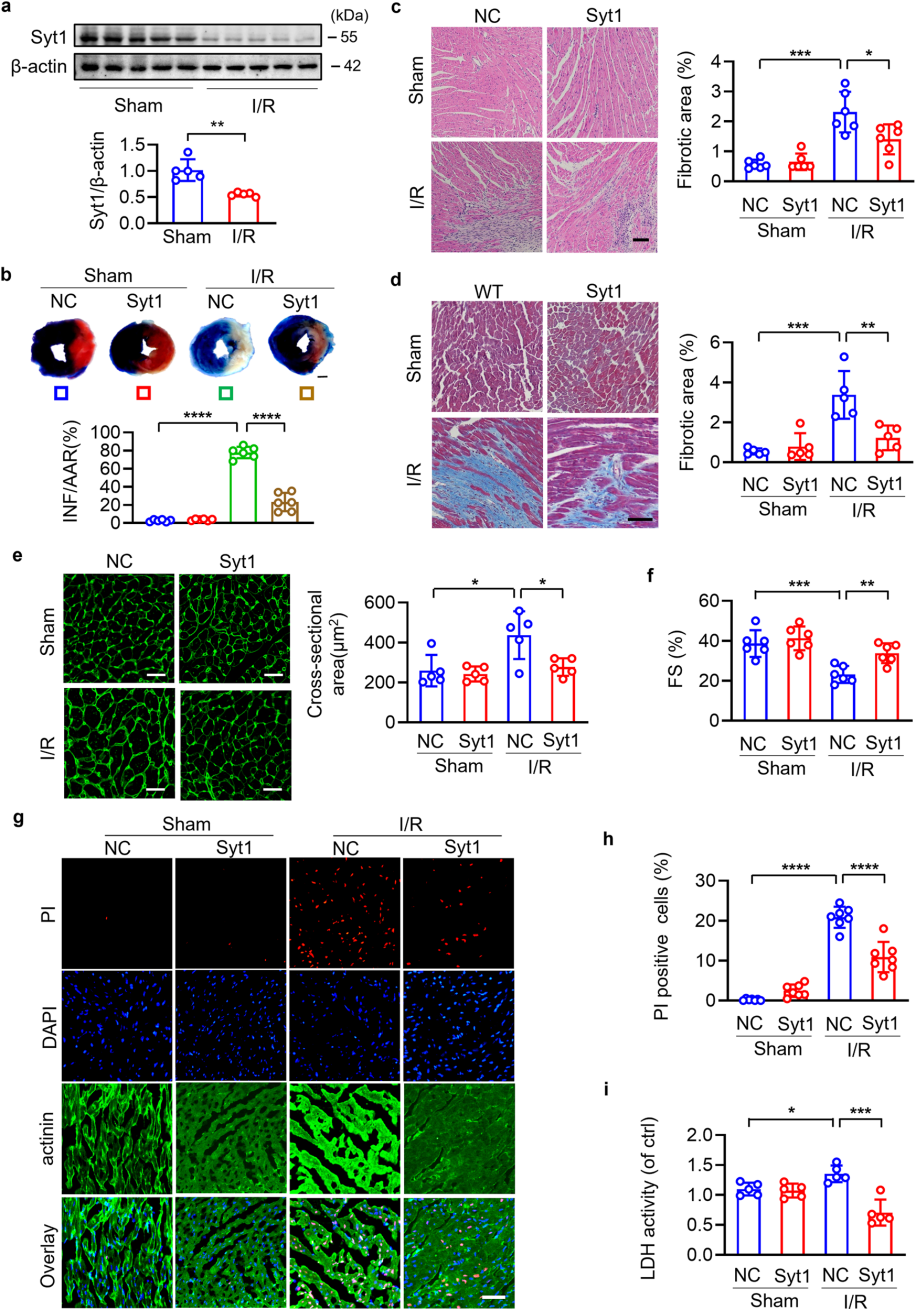
Syt1 protects hearts against cardiomyocyte necrosis and ischemia/reperfusion (I/R) injury. **a** Mice hearts were subjected to I/R or sham surgery. Then the protein level of Syt1 in the LV was detected by western blotting. ** *P* < 0.01. N = 5. **b**–**i** Hearts were injected with Syt1 overexpression adenovirus (Syt1) or negative control (NC), followed by I/R or sham surgery. **b** Evens blue/TTC staining was performed to assess infarct size. The blue zone represents non-risk area, the off-white zone represents infarct area (INF). The infarct size was calculated by the ratio of infarct area (INF) to area at risk (AAR) (off-white plus red). Bar = 2.5 mm. **** *P* < 0.0001. N = 6. **c**, **d** The fibrotic area was analyzed by HE staining (N = 6) and Masson trichrome staining (N = 5). * *P* < 0.05. ** *P* < 0.01. *** *P* < 0.001. Bar = 100 µm. **e** TRITC-conjugated wheat germ agglutinin (WGA) staining was performed to analyze the cross-sectional area of cardiomyocytes. Bar = 25 µm. * *P* < 0.05. N = 5. **f** Fractional shortening (FS) of LV. ** *P* < 0.01. *** *P* < 0.001. N = 6. **g**–**i** Necrotic cell death detection. PI staining was performed. Red, PI; blue, DAPI; green, actinin. Bar = 50 µm. **** *P* < 0.0001. N = 7. Lactate dehydrogenase (LDH) activity. * *p* < 0.05. *** *p* < 0.001. N = 5. These data are expressed as the mean ± SD.

Syt1 has been demonstrated to express in the heart, however, whether it functions in cardiac pathology remains unknown [27, 31]. We first detected the expression levels of Syt1 in I/R injured hearts. Results showed that Syt1 were significantly downregulated upon I/R (Fig. 1a). To investigate the role of Syt1 in I/R injury, Syt1-overexpressing adenovirus was injected into in situ hearts (Fig. S1a). Syt1-overexpressing mice showed significantly decreased infarct area assessed by evens blue/TTC staining (Fig. 1b) and suppressed interstitial fibrosis examined by hematoxylin-eosin staining (Fig. 1c) and Masson’s trichrome staining (Fig. 1d), compared to wide-type mice subjected to I/R injury. WGA is widely used for cell membrane presentation utilizing its property that recognizes N-acetylglucosamine in cell membrane. Cell size could be assessed by WGA staining. Attenuated cardiomyocytes size enlargement (Fig. 1e) and improved cardiac function (Fig. 1f) were also observed in infarct hearts with Syt1 overexpression. By performing Propidium iodide (PI)-staining and lactate dehydrogenase (LDH) activity assays, we analyzed cardiac cell death in response to MI. Necrosis was observed in infarct areas, which was significantly attenuated by overexpressing Syt1 (Fig. 1g−i). These results indicate that Syt1 mitigates myocardial necrosis and I/R injury.

### Synaptotagmin-1 inhibits cardiomyocyte programmed necrosis and mPTP opening under oxidative stress

**Fig. 2:**
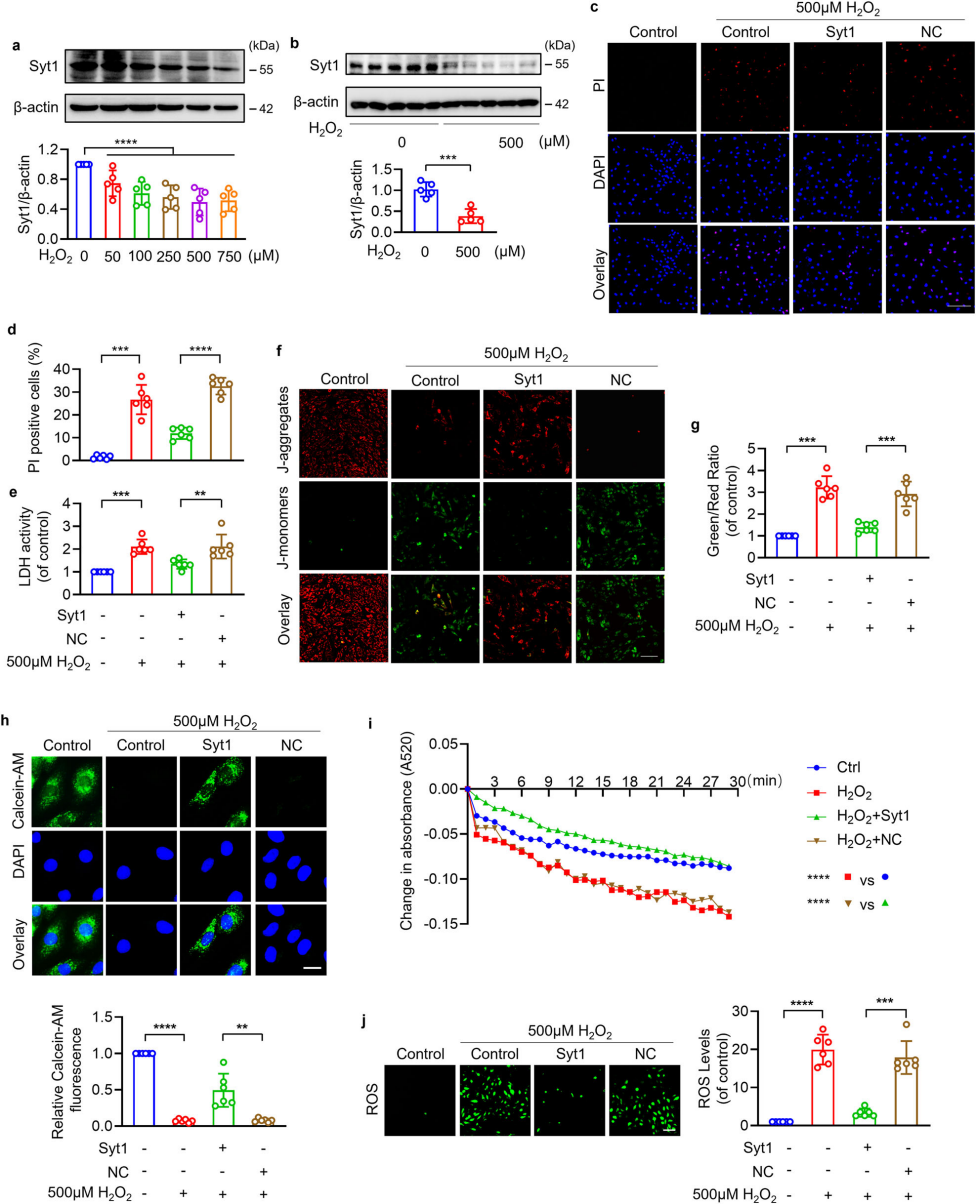
Syt1 inhibits H_2_O_2_-induced cardiomyocyte necrosis and mPTP opening. **a**, **b** Western blotting showing the protein levels of Syt1 in cardiomyocytes exposed to H_2_O_2_ at the indicated concentration. *** *P* < 0.001. **** *P* < 0.0001. N = 5. **c**–**e** Necrotic cell death detection. Cardiomyocytes were infected with Syt1 overexpression adenovirus (Syt1) or negative control (NC), and then treated with 500 μM H_2_O_2_. PI staining was performed to indicate necrotic cell death. Bar = 100 µm. *** *P* < 0.001. **** *P* < 0.0001. N = 6. LDH activity was analyzed. ** *P* < 0.01. *** *P* < 0.001. N = 6. **f**, **g** Analysis of mitochondrial membrane potential. The 5,5′,6,6**′**-Tetrachloro-1,1′,3,3′-tetraethyl-imidacarbocyanine iodide (JC-1) was used to analyze the mitochondrial membrane potential △Ψm. The ratio of J-monomers/J-aggregates was calculated. Bar = 100 µm. *** *P* < 0.001. N = 6. **h**, **i** mPTP opening analysis. **h** The Calcein-AM fluorescence intensity was detected in cardiomyocytes infected with Syt1 overexpression adenovirus (Syt1) or negative control (NC), and then treated with 500 μM H_2_O_2_. Bar = 50 µm. ** *P* < 0.01. **** *P* < 0.0001. N = 6. **i** Mitochondrial swelling was analyzed by monitoring the absorbance at 520 nm every 1 min for a total of 30 min. **** *P* < 0.0001. N = 6. **j** ROS was detected by fluorescent probe DCFH-DA. Bar = 100 µm. *** *P* < 0.001. **** *P* < 0.0001. N = 6. These data are expressed as the mean ± SD. These data are expressed as the mean ± SD.

We further investigated the role of Syt1 in cardiac oxidative stress injury at a cellular level and the underlying mechanisms. H_2_O_2_ has been widely used as an oxidant to induce oxidative stress. Our previous work has demonstrated that cardiomyocyte apoptotic cell death was mainly induced by low dose of H_2_O_2_, and necrotic cell death was triggered by high dose of H_2_O_2_ [5, 15]. We showed here that Syt1 was downregulated in H_2_O_2_-treated cardiomyocytes in a concentration-dependent manner (Fig. 2a), and higher concentration of H_2_O_2_ induced a significant decrease of Syt1 (Fig. 2a, b), suggesting that Syt1 participates in H_2_O_2_-induced cardiomyocyte necrosis. PI staining was performed and the necrotic cells were measured by PI incorporation. Increased necrotic cells and enhanced LDH activity were observed in cardiomyocytes exposed to 500 μM H_2_O_2_, which was attenuated by Syt1 overexpression (Fig. 2c–e). Knockdown of endogenous Syt1 sensitized cardiomyocytes to necrosis upon 100 μM H_2_O_2_ treatment (Figs. S1b, c and S2a–c). These results indicate that Syt1 negatively regulates H_2_O_2_-induced cardiac necrosis.

Because prolonged opening of mPTP mediates the mitochondrial regulatory pathway of programmed necrosis [12, 14], we next analyzed whether Syt1 was involved in mPTP opening. The 5,5′,6,6′-Tetrachloro-1,1′,3,3′-tetraethyl-imidacarbocyanine iodide (JC-1) is a fluorescent probe to detect mitochondrial membrane potential △Ψm and is widely used to predict mPTP excessive opening. JC-1 aggregates in the matrix of mitochondria to form the polymer (J-aggregates) when the mitochondrial membrane potential is high; while JC-1, as a monomer (J-monomers), cannot aggregate in the matrix of mitochondrial when the mitochondrial membrane potential is low. Because J-aggregates and J-monomers emit at different wavelengths, they could be detected separately [33]. We found that J-aggregates were reduced and J-monomers were increased in cardiomyocytes exposed to 500 μM H_2_O_2_, which was significantly rescued by Syt1 overexpression (Fig. 2f, g). Inhibition of endogenous Syt1 sensitized 100 μM H_2_O_2_-treated cardiomyocytes to △Ψ dissipation (Fig. S2d, e). To further confirm the role of Syt1 on the mPTP dynamics, we utilized Calcein-AM staining and mitochondrial swelling assay to evaluate mPTP opening [8, 34–36]. Calcein-AM can pass through mitochondrial membranes and emit green fluorescence which could not be quenched by CoCl_2_, as CoCl_2_ could not cross the normal mitochondrial membrane. However, when mPTP opens, CoCl_2_ enters mitochondria and then quenches the fluorescence of Calcein-AM. The mPTP opening was determined by the reduction in calcein fluorescence in the mitochondria [35, 36]. Our results showed that enforced expression of Syt1 significantly attenuated 500 μM H_2_O_2_-induced loss of calcein fluorescence (Fig. 2h) and mitochondrial swelling (Fig. 2i), while knockdown of Syt1 sensitized 100 μM H_2_O_2_-treated cardiomyocytes to loss of calcein fluorescence (Fig. S2f) and mitochondrial swelling (Fig. S2g). Additionally, Syt1 suppressed ROS production induced by H_2_O_2_ (Figs. 2j and S2h). These results indicate that Syt1 inhibited H_2_O_2_-induced mPTP excessive opening.

Hypoxia/reoxygenation (H/R)- induced cardiomyocyte injury model was used to further evaluate the function of Syt1 in cardiomyocyte necroptosis. Cardiomyocytes exhibited a significantly decreased Syt1 upon H/R treatment (Fig. S3a). Enforced expression of Syt1 significantly suppressed H/R-induced necrotic cell death assessed by PI incorporation and LDH activity (Fig. S3b–d). H/R triggered mitochondrial membrane potential dissipation (Fig. S3e, f) and mPTP prolonged opening (Fig. S3g), which was attenuated by Syt1 overexpression. In summary, these data suggest that Syt1 regulates programmed necrosis and mPTP opening in cardiomyocytes under oxidative stress.

### Synaptotagmin-1 is required for Parkin to catalyze ubiquitination of CypD

**Fig. 3:**
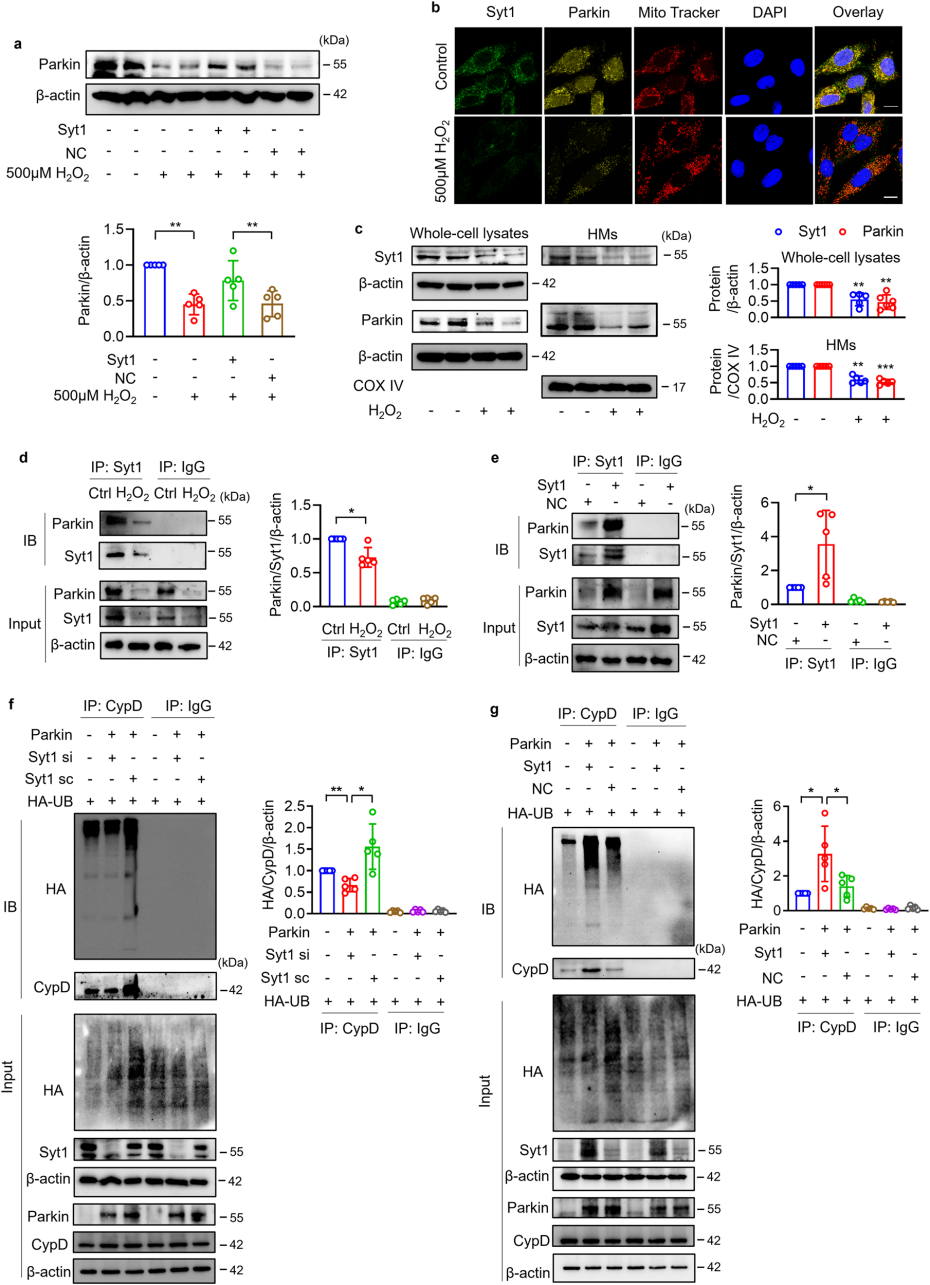
Interaction between Syt1 and Parkin. **a** Western blotting showing the protein levels of Parkin. Cardiomyocytes were infected with Syt1 overexpression adenovirus (Syt1) or negative control (NC), and then treated with 500 μM H_2_O_2_. ** *P* < 0.01. N = 5. **b**, **c** The cellular and subcellular localization analysis of Syt1 and Parkin. **b** Immunofluorescent staining to visualize the distributions of Syt1 and Parkin. Cardiomyocytes were stained with MitoTracker (red), followed by incubated with anti-Syt1 antibody and anti-Parkin antibody. Green, Syt1; yellow, Parkin; red, mitochondria; Blue, DAPI. Bar = 15 µm. **c** Protein expression levels of Syt1 and Parkin in whole-cell lysates or mitochondria-enriched heavy membranes (HMs) exposed to H_2_O_2_. ** *P* < 0.01. *** *P* < 0.001. N = 5, 6. **d**, **e** Immunoprecipitation showing th**e** interaction between Parkin and Syt1. Syt1, Syt1 overexpression adenovirus. NC, negative control adenovirus. * *P* < 0.05. N = 5. **f**, **g** Effect of Syt1 on Parkin-catalyzed ubiquitination of CypD. After transfected with expression vectors encoding HA-ubiquitin (HA-UB), HEK293 cells were infected with Syt1 siRNAs adenovirus or Syt1 overexpression adenovirus, followed by infected with Parkin overexpression adenovirus. Then immunoprecipitation was performed to detected the ubiquitination levels of CypD. Parkin, Parkin overexpression adenovirus; Syt1 si, Syt1 siRNAs adenovirus; Syt1 sc, scrambled Syt1 adenovirus; Syt1, Syt1 overexpression adenovirus. * *P* < 0.05. ** *P* < 0.01. N = 5. These data are expressed as the mean ± SD.

CypD is a core component of mPTP and critically regulates mPTP opening [37, 38]. Our previous work has demonstrated the Parkin-CypD-mPTP axis as an important necrosis regulation pathway in myocardial ischemia/reperfusion injury. Parkin interacts with CypD and catalyzes CypD ubiquitination. Under moderate oxidative stress, Parkin is translocated to mitochondria and inhibits mPTP opening by targeting CypD [15]. Here, Parkin was downregulated in 500 μM H_2_O_2_-treated cardiomyocytes, which was rescued by enforced expression of Syt1 (Fig. 3a). Therefore, we speculated that Syt1 may interact with Parkin. Co-localization of Syt1 and Parkin in mitochondria was observed, while H_2_O_2_ decreased the mitochondrial accumulation of both Syt1 and Parkin (Fig. 3b, c). IP results showed that Parkin was immunoprecipitated by Syt1, and this interaction was attenuated by 500 μM H_2_O_2_ treatment (Fig. 3d). Overexpression of Syt1 induced a significant increase in the immunoprecipitated Parkin (Fig. 3e). We further investigated whether Syt1 is involved in Parkin-catalyzed CypD ubiquitination. Increased ubiquitinated CypD protein was detected upon enforced expression of Parkin, which was attenuated by knockdown of Syt1 (Fig. 3f). Overexpression of Syt1 significantly augmented the catalytic effect of Parkin on CypD ubiquitination (Fig. 3g). Taken together, this part of experiments demonstrates that Syt1 interacts with Parkin and is required for Parkin to catalyze CypD ubiquitination.

### Synaptotagmin-1 suppresses cardiomyocyte necrosis and mPTP opening by targeting Parkin

**Fig. 4:**
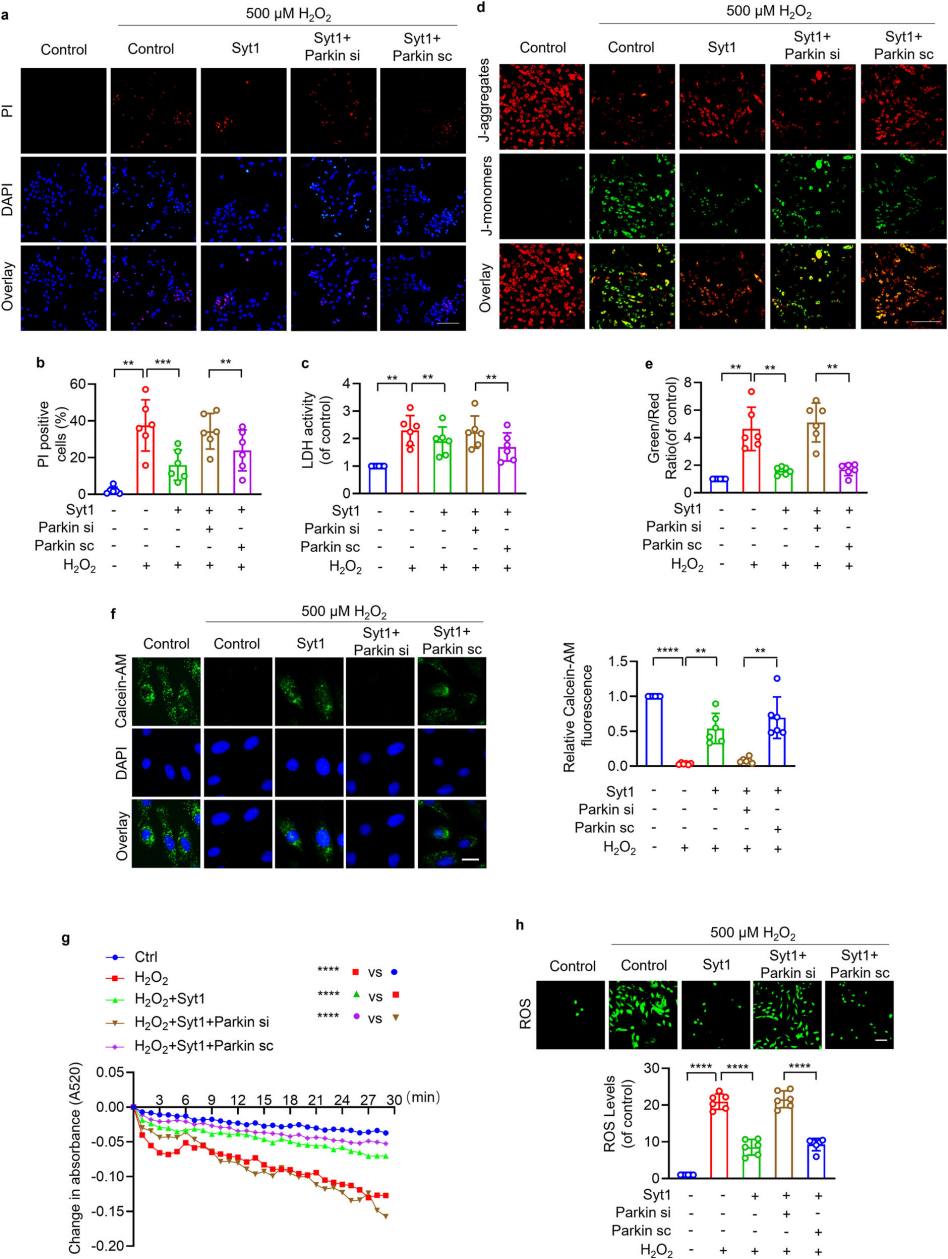
Syt1-regulated cardiomyocyte necrosis and mPTP opening by targeting Parkin. Cardiomyocytes were infected with Parkin siRNAs adenovirus (Parkin si) or scrambled Parkin adenovirus (Parkin sc), followed by infected with Syt1 adenovirus (Syt1). Then the cells were exposed to 500 µm H_2_O_2_ for 12 h. **a**, **b** PI staining. Bar = 100 µm. ** *P* < 0.01. *** *P* < 0.001. N = 6. **c** LDH activity detection. ** *P* < 0.01. N = 6. **d**, **e** JC-1 was used to analyze the mitochondrial membrane potential △Ψm. The ratio of J-monomers/J-aggregates was calculated. Bar = 100 µm. ** *P* < 0.01. N = 6. **f**, **g** mPTP opening analysis. **f** The calcein-AM fluorescence intensity detection. Bar = 50 µm. ** *P* < 0.01. **** *P* < 0.0001. N = 6. **g** Mitochondrial swelling was analyzed by monitoring the absorbance at 520 nm every 1 min for a total of 30 min. **** *P* < 0.0001. N = 6. **h** ROS was detected by fluorescent probe DCFH-DA. Bar = 100 µm. **** *P* < 0.0001. N = 6. These data are expressed as the mean ± SD.

Next, we explored whether Syt1 suppressed mPTP opening and cardiomyocyte programmed necrosis through Parkin. High concentration of H_2_O_2_ increased PI-positive cardiomyocytes and LDH activity, which was attenuated by Syt1 overexpression, while this inhibitory effect of Syt1 was diminished by knocking down Parkin (Fig. 4a–c). Enforced expression of Syt1 rescued H_2_O_2_-induced mitochondrial potential dissipation (Fig. 4d, e), loss of calcein-AM fluorescence (Fig. 4f), mitochondrial swelling (Fig. 4g) and ROS production (Fig. 4h), which was attenuated by inhibition of endogenous Parkin. These results indicate that Syt1 represses cardiomyocyte programmed necrosis and mPTP opening by targeting Parkin.

### Synaptotagmin-1 is suppressed by miR-193b-3p

**Fig. 5:**
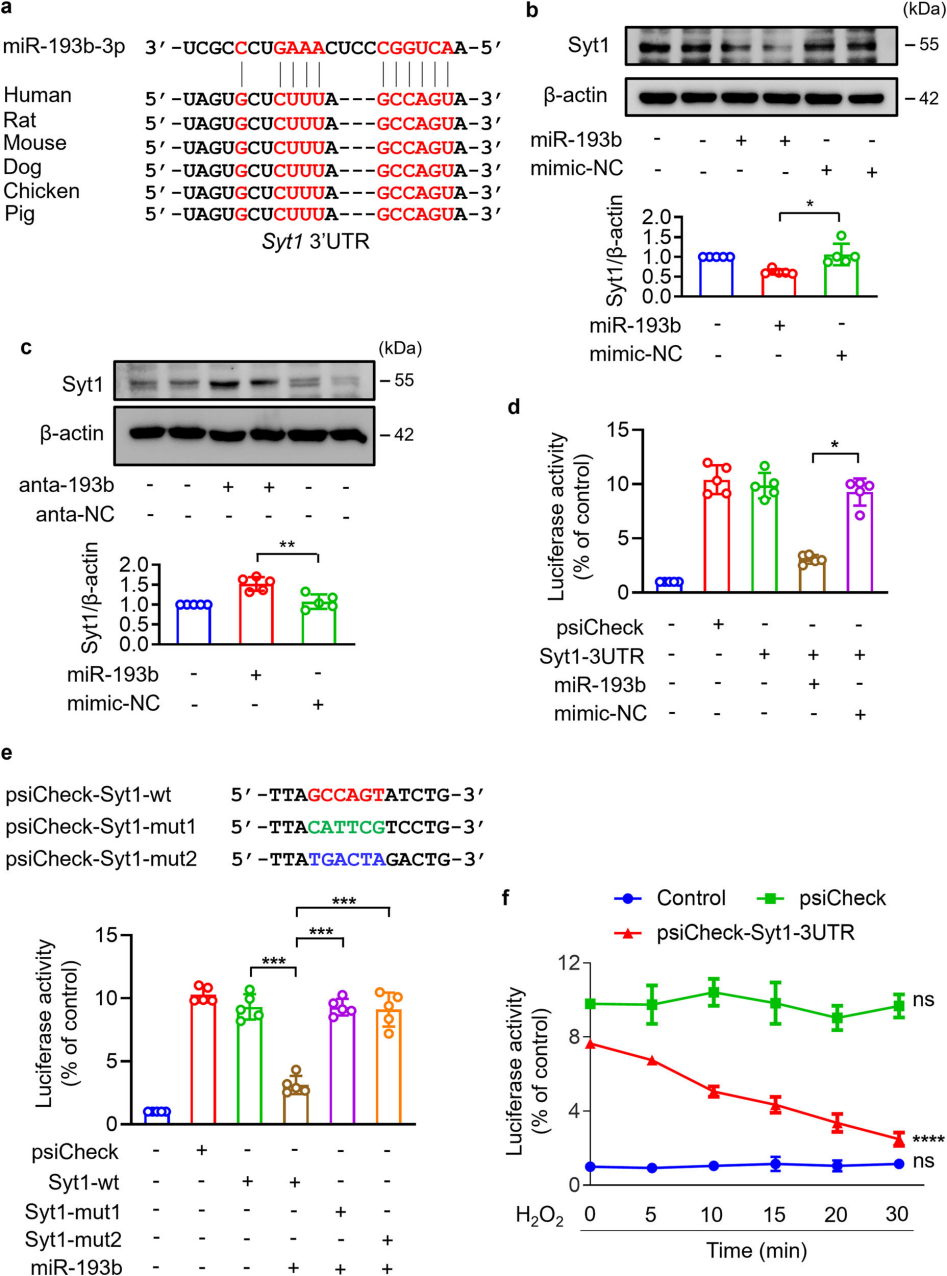
miR-193b-3p targeted Syt1 and suppressed Syt1 expression. **a** Putative binding sites of miR-193b-3p in the 3′UTR of Syt1. **b**, **c** Syt1 protein level in cardiomyocytes transfected with miR-193b-3p mimic (miR-193b) or antagomir (anta-193b). anta-NC, negative control of antagomir; mimic-NC, negative control of mimic. **d**, **e** The interaction analysis between miR-193b-3p and Syt1. The 3′UTR of Syt1 mRNA or two types of mutated 3′UTR were cloned into dual-luciferase reporter gene vector psiCHECK-2, respectively. HEK293 cells were transfected with psiCHECK-2 vectors and miR-193b-3p mimic. Then the activities of firefly luciferase and renilla luciferase were measured. * *P* < 0.05. *** *P* < 0.001. N = 5. **f** Effect of H_2_O_2_ on interaction between miR-193b-3p and Syt1. Cardiomyocytes were transfected with psiCHECK-2 vectors expressing Syt1 3′UTR, followed by exposed to 500 µM H_2_O_2_ for the indicated time. Then the activities of firefly luciferase and renilla luciferase were measured. **** *P* < 0.0001. ns: no statistical significance. N = 5. These data are expressed as the mean ± SD.

The upstream regulatory mechanisms involved in Syt1-regulated cardiac programmed necrosis was further investigated. The sequence of *Syt1* mRNA was analyzed by the bioinformatic program TargetScan, and twenty-odd complementary sequences of microRNAs (miRs) were identified. Among them only the binding site for miR-193b-3p and miR-193a-3p was putative conserved and was identified in the 3′UTR of *Syt1* mRNA (Fig. 5a). It has been reported that miR-193b-3p participated in the cardiac pathologic regulation, while the role of miR-193a-3p in cardiovascular system remains unclear. Therefore, miR-193b-3p was chosen as potential regulator of Syt1, and further experiment was performed. Overexpression of miR-193b-3p decreased Syt1 protein level (Fig. 5b), while knockdown of miR-193b-3p increased Syt1 protein level (Fig. 5c). To clarify the interaction between miR-193b-3p and Syt1, dual-luciferase assay was performed. The 3′UTR of Syt1 mRNA containing miR-193b-3p potential binding sites or mutated binding sites were cloned into psiCHECK-2 luciferase reporter gene vector (Fig. 5d−f). Compared to the control cells, HEK293 cells transfected with psiCHECK-2 vector cloned with *Syt1* 3′UTR showed increased chemiluminescent signal, which was significantly attenuated by enforced expression of miR-193b-3p (Fig. 5d). MiR-193b-3p inhibited the increasing of chemiluminescent signal induced by psiCHECK-2 vector cloned with *syt1* 3′UTR, but this inhibitory effect was canceled upon mutating the potential binding sites (Fig. 5e). These results indicate that miR-193b-3p suppresses Syt1 expression by directly binding to the predicted sites of *Syt1* 3′UTR. In addition, we analyzed the interaction between miR-193b-3p and Syt1 in cardiomyocytes. A time-dependent decrease of the chemiluminescent signal was observed from cardiomyocytes transfected with psiCHECK-2 vector cloned with *syt1* 3′UTR, compared with that of control group or psiCHECK-2 empty vector group (Fig. 5f). Taken together, this part of experiments demonstrates that miR-193b-3p directly binds to the 3′UTR of *Syt1* mRNA and inhibits its expression.

### MiR-193b-3p regulates cardiac necrosis and I/R injury

**Fig. 6:**
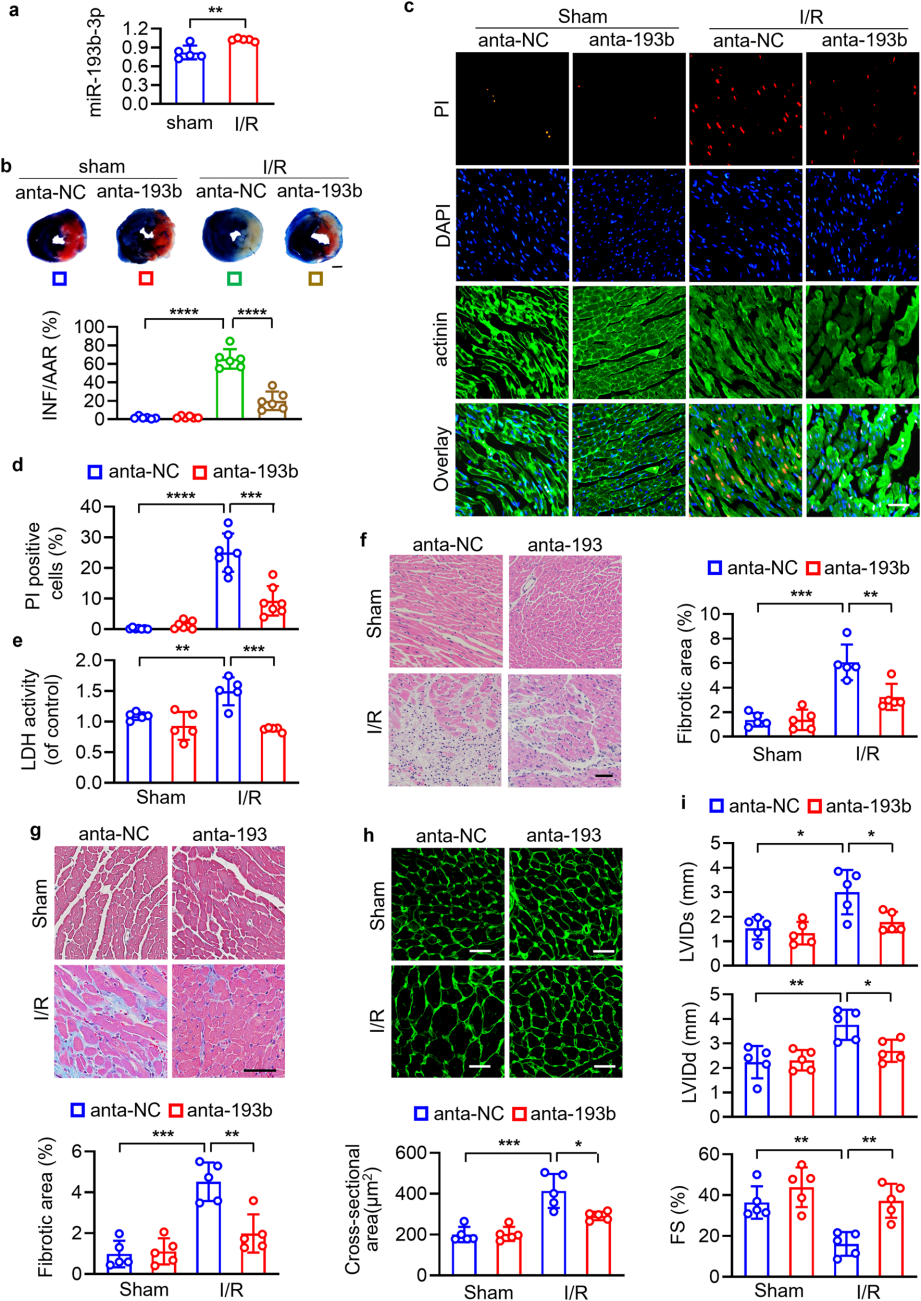
miR-193b-3p regulated cardiomyocyte necrosis and I/R injury. **a** Detection of miR-193b-3p levels in hearts subjected to I/R or sham surgery by qRT-PCR. ** *P* < 0.01. N = 5. **b**–**i** Hearts were injected with miR-193b-3p antagomir adenovirus (anta-193b) or negative control (anta-NC), followed by I/R or sham surgery. **b** Evens blue/TTC staining. The blue zone represents non-risk area, the off-white zone represents infarct area (INF). The infarct size was calculated by the ratio of infarct area (INF) to area at risk (AAR) (off-white plus red). Bar = 2.5 mm. **** *P* < 0.0001. N = 6. **c**–**e** Necrotic cell death detection. PI staining was performed. Red, PI; blue, DAPI; green, actinin. Bar = 50 µm. *** *P* < 0.001. **** *P* < 0.0001. N = 7. Lactate dehydrogenase (LDH) activity was detected. ** *P* < 0.01. *** *P* < 0.001. N = 5. **f**, **g** The fibrotic area was analyzed by HE staining (N = 5) and Masson trichrome staining (N = 5). ** *P* < 0.01. *** *P* < 0.001. Bar = 50 µm. **h** TRITC-conjugated wheat germ agglutinin (WGA) staining was performed to analyze the cross-sectional area of cardiomyocytes. * *P* < 0.05. *** *P* < 0.001. N = 5. Bar = 25 µm. **i** LV systolic internal diameters (LVIDs), LV diastolic internal diameters (LVIDd) and LV Fractional shortening (FS). * *P* < 0.05. ** *P* < 0.05. N = 5. These data are expressed as the mean ± SD.

Recent advances have revealed that miR-193b-3p participates in the regulation of pulmonary hypertension in heart failure [39], cardiac hypertrophy [40] and apoptotic pathway in myocardial I/R injury [41]. However, the role of miR-193b-3p in cardiac programmed necrosis has not been clarified. Here we found that miR-193b-3p was significantly upregulated upon I/R injury (Fig. 6a). Knockdown of miR-193b-3p significantly reduced the infarct area (Figs. 6b and S4a). Knockdown of miR-193b-3p inhibited cardiomyocyte necrotic cell death and decreased LDH activity (Fig. 6c−e). Additionally, antagonizing miR-193b-3p by its antagomirs attenuated I/R-induced interstitial fibrosis as assessed by hematoxylin-eosin staining (Fig. 6f) and Masson’s trichrome staining (Fig. 6g). Wheat germ agglutinin (WGA) staining was performed to assess cardiomyocyte size, and echocardiography was performed to analyze cardiac function. The surface area of cardiomyocytes indicating cell size was increased in mice with I/R, which was attenuated by miR-193b-3p antagomir (Fig. 6h). Knockdown of miR-193b-3p decreased the left ventricular systolic internal diameter (LVIDs) and left ventricular diastolic internal diameter (LVIDd), but increased the fraction shortening (FS) (Fig. 6i). These results indicate that inhibition of miR-193-3p prevents the necrotic cell death, cardiac damage and cardiac dysfunction induced by I/R.

### MiR-193b-3p mediates cardiomyocyte necrosis and mPTP opening by targeting Syt1

**Fig. 7:**
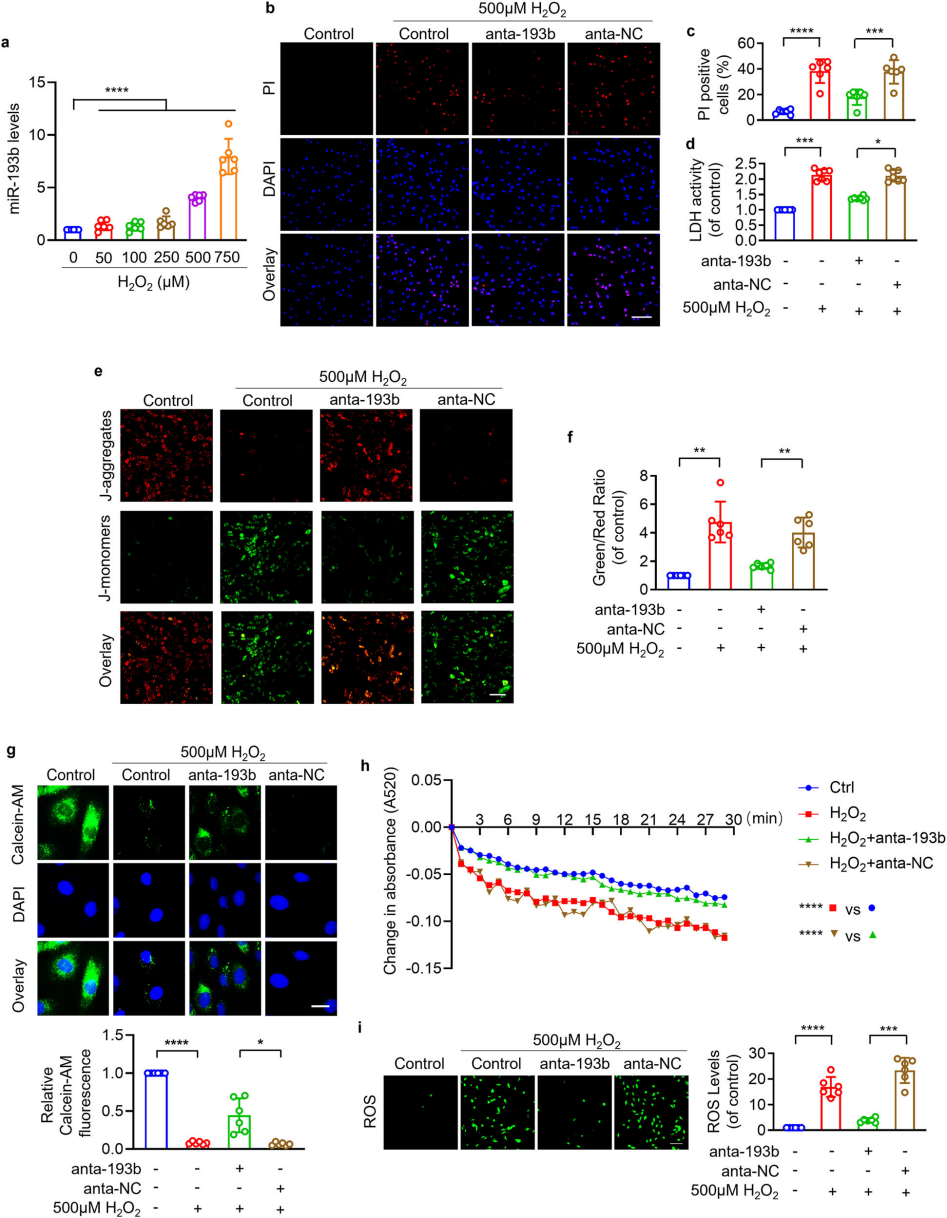
miR-193b-3p promoted cardiomyocyte necrosis and mPTP opening by targeting Syt1. **a** The levels of miR-193b-3p were detected in cardiomyocytes treated with H_2_O_2_ at the indicated concentration. **** *P* < 0.0001. N = 6. **b**–**d** Necrotic cell death analysis. Cardiomyocytes were infected with miR-193b-3p antagomir adenovirus (anta-193b) or negative control (anta-NC), and then treated with 500 μM H_2_O_2_. PI staining was performed to indicate necrotic cell death. Bar = 50 µm. *** *P* < 0.001. **** *P* < 0.0001. N = 6. LDH activity was analyzed. * *P* < 0.05. *** *P* < 0.001. N = 6. **e**, **f** Mitochondrial membrane potential detection by JC-1 staining. The ratio of J-monomers/J-aggregates was calculated. Bar = 100 µm. ** *P* < 0.01. N = 6. These data are expressed as the mean ± SD. **g**, **h** mPTP opening evaluation. **g** The calcein-AM fluorescence intensity detection. Bar = 50 µm. * *P* < 0.05. **** *P* < 0.0001. N = 6. **h** Mitochondrial swelling was analyzed by monitoring the absorbance at 520 nm every 1 min for a total of 30 min. **** *P* < 0.0001. N = 6. **i** ROS was detected by fluorescent probe DCFH-DA. Bar = 100 µm. *** *P* < 0.001. **** *P* < 0.0001. N = 6. These data are expressed as the mean ± SD.

Next, we explored the role of miR-193b-3p in H_2_O_2_-induced cardiomyocyte programmed necrosis and mPTP opening. MiR-193b-3p accumulated in cardiomyocyte upon H_2_O_2_ challenge in a concentration-dependent manner (Fig. 7a). Cardiomyocytes with less miR-193b-3p exhibited attenuated necrotic cell death and LDH activity in response to 500 μM H_2_O_2_ (Figs. 7b−d and S4b), while cardiomyocytes with more miR-193b-3p were sensitive to necrosis upon 100 μM H_2_O_2_ treatment (Figs. S4c and S5a−c). Mitochondrial membrane potential was dissipated in cardiomyocytes exposed to 500 μM H_2_O_2_, which was rescued by knockdown of miR-193b (Fig. 7e, f). Overexpression of miR-193b-3p sensitized 100 μM H_2_O_2_-treated cardiomyocytes to mitochondrial potential dissipation demonstrated by increased J-monomers and decreased J-aggregates (Fig. S5d, e). Antagonizing miR-193b-3p significantly attenuated mitochondrial potential dissipation (Fig. 7e, f), delayed the calcein fluorescence loss (Fig. 7g), suppressed mitochondrial swelling (Fig. 7h), and decreased ROS levels (Fig. 7i). Cardiomyocytes transfected with miR-193b-3p mimics were sensitized to mPTP opening (Fig. S5f, g) and ROS accumulation (Fig. S5h) in response to 100 μM H_2_O_2_. Additionally, the role of Syt1 in cardiomyocyte necrosis and mPTP opening was further evaluated in H/R model. MiR-193b-3p was upregulated in cardiomyocytes exposed to H/R (Fig. S6a). Antagonizing miR-193b-3p significantly reduced PI-positive cells (Fig. S6b, c) and decreased LDH activity (Fig. S6d) in H/R-treated cardiomyocytes. H/R-induced mitochondrial membrane potential dissipation (Fig. S6e, f) and mPTP prolonged opening (Fig. S6g) was rescued by inhibition of miR-193b-3p. Taken together, we conclude that miR-193b-3p mediates cardiomyocyte necrosis and mPTP opening under oxidative stress.

**Fig. 8:**
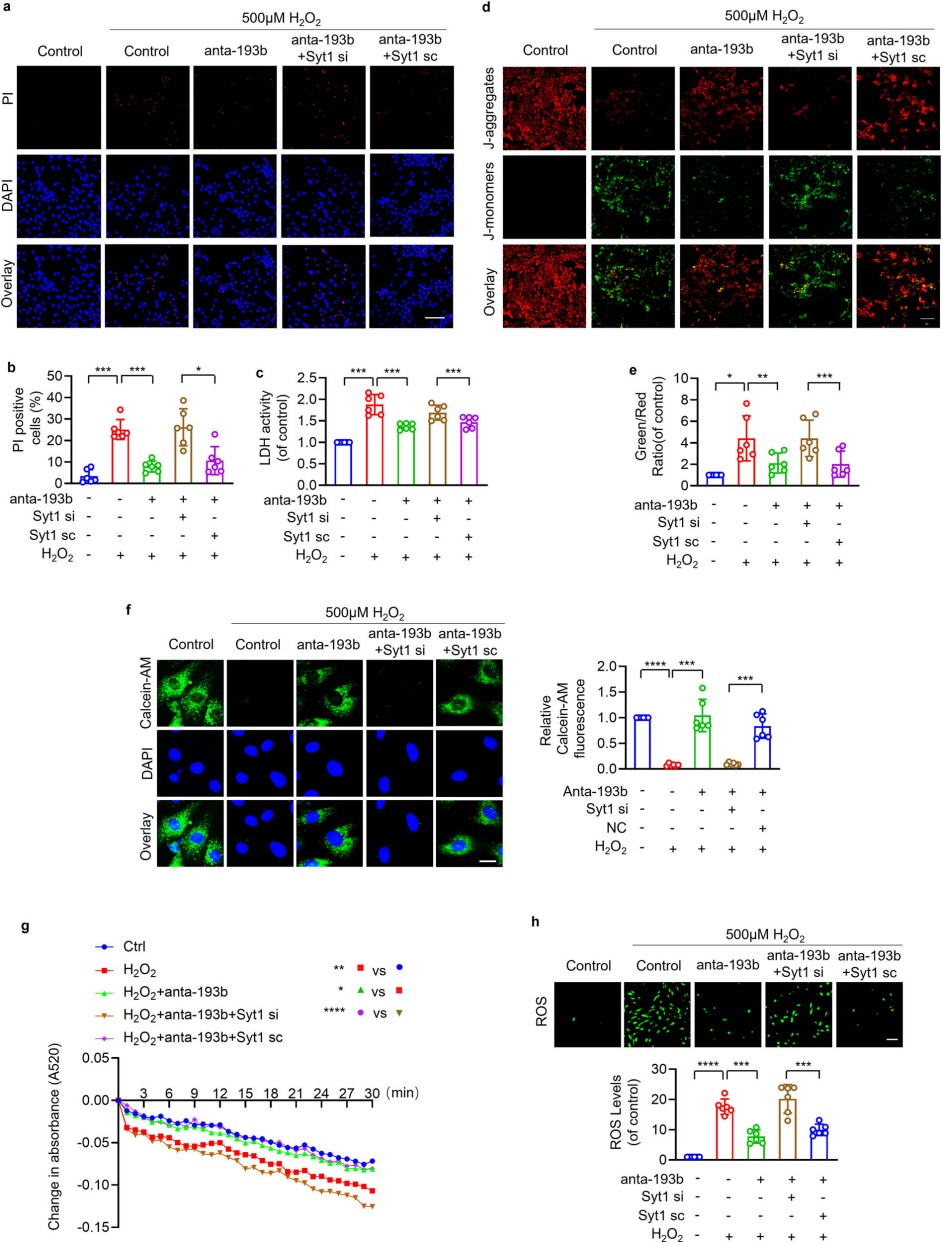
miR-193b-3p regulated cardiomyocyte necrosis and mPTP opening through Syt1. Cardiomyocytes were infected with Syt1 siRNA adenovirus (Syt1 si) or scrambled Syt1 adenovirus (Syt1 sc), followed by infected with miR-193b-3p antagomir adenovirus (anta-193b). Then the cells were exposed to 500 µm H_2_O_2_ for 12 h. **a**, **b** PI staining was performed. Bar = 50 µm. * *P* < 0.05. *** *P* < 0.001. N = 6. **c** LDH activity was measured. *** *P* < 0.001. N = 6. **d**, **e** JC-1 was used to detect mitochondrial membrane potential △Ψm. The ratio of J-monomers/J-aggregates was calculated. Bar = 100 µm. * *P* < 0.05. ** *P* < 0.01. *** *P* < 0.001. N = 6. These data are expressed as the mean ± SD. **f**, **g** mPTP opening analysis. **f** The calcein-AM fluorescence intensity detection. Bar = 50 µm. *** *P* < 0.001. **** *P* < 0.0001. N = 6. **g** Mitochondrial swelling was analyzed by monitoring the absorbance at 520 nm every 1 min for a total of 30 min. * *P* < 0.05. ** *P* < 0.01. **** *P* < 0.0001. N = 6. **h** ROS was detected by fluorescent probe DCFH-DA. Bar = 100 µm. *** *P* < 0.001. **** *P* < 0.0001. N = 6. These data are expressed as the mean ± SD.

We further explored whether miR-193b-3p regulated cardiomyocyte programmed necrosis through targeting Syt1. Results showed that antagonizing miR-193b-3p by its antagomir attenuated 500 μM H_2_O_2_-induced necrotic cell death and LDH activity, which was abolished by inhibition of Syt1 (Fig. 8a−c). The inhibitory effect of miR-193b-3p antagomir on mitochondrial membrane potential dissipation (Fig. 8d, e), loss of calcein fluorescence (Fig. 8f), mitochondrial swelling (Fig. 8g), and ROS accumulation (Fig. 8h) was attenuated by knockdown of Syt1. Taken together, we conclude that miR-193b-3p and Syt1 constitute a regulatory axis for programmed cardiomyocyte necrosis and mPTP opening.

## Discussion

Programmed necrosis greatly contributes to the pathogenesis of cardiac disorders, especially severe myocardial damage such as I/R injury. Our present work identified that Syt1 was an anti-necrosis and anti-I/R injury regulator. Syt1 inhibited I/R-induced necrotic cell death and I/R injury, and improved cardiac function. Syt1 was downregulated under oxidative stress, and overexpression of Syt1 suppressed H_2_O_2_- and H/R- induced cardiomyocyte necrosis and mPTP opening. In further exploring the underlying mechanisms, we found that Syt1 interacted with Parkin and was required for Parkin to catalyze CypD ubiquitination. Syt1-regulated cardiomyocyte necrosis by targeting the Parkin-CypD-mPTP pathway. In addition, Syt1 expression was suppressed by miR-193b-3p. MiR-193b-3p mediated cardiomyocyte necrosis both in vivo and in vitro, and this regulatory effect was through targeting Syt1. Briefly, our work provides new insights into understanding the pathogenesis of I/R injury, and reveals a novel regulatory model of cardiomyocyte necrosis composed of miR-193b-3p, Syt1, Parkin, CypD and mPTP.

Syt1 was originally identified in the nervous system and functions in neurotransmitters release from presynaptic nerve terminals [16–21]. Further studies have demonstrated that Syt1 is expressed in some tumor tissues and tumor cells including thyroid cancer tissues, head and neck squamous cell carcinoma (HNSCC) tissues [42, 43]. Syt1 is also expressed in mouse islets tissues, insulinoma cell lines, mast cells from mucosal and connective tissues. In 2006, Syt1 was reported distributed in atrial cardiomyocytes from neonatal and adult rats [27]. In the present study, we investigated the expressions and functions of Syt1 in the left ventricle (LV) and cardiomyocyte. We found that Syt1 is highly expressed in the LV tissues of mouse hearts and H9c2 cells, and was significantly downregulated upon I/R, H_2_O_2_ and H/R challenge. The location of Syt1 in mitochondria was also identified. The wide distribution of Syt1 in cardiomyocytes and the significant expression change under oxidative stress suggests its potential role in pathogenesis of cardiac disorders. Our data revealed that Syt1 protected hearts against programmed necrosis and I/R injury. Enforced expression of Syt1 attenuated cardiac I/R injury, interstitial fibrosis, necrotic cell death and improved cardiac function.

A mass of reactive oxygen species (ROS) is generated in hearts with I/R, which directly injured the myocardium followed by cardiodepression. The burst release of ROS through prolonged mPTP opening results in dissipation of mitochondrial membrane potential, organelle swelling and rupture, mitochondrial dysfunction and cell death [8, 9, 44]. Therefore, myocardial I/R model, cardiomyocyte H/R model and H_2_O_2_-damaged cardiomyocyte model were constructed to study myocardial oxidative stress injury. Syt1 has been reported to be associated with oxidative stress regulation in glioma, neuron, and diabetic encephalopathy, but direct evidence is lacking [28–30]. Here we demonstrated that Syt1 was abundantly distributed in the mitochondria of cardiomyocytes. Enforced expression of Syt1 significantly inhibited mPTP abnormal opening and mitochondria-dependent necrotic cell death in response to oxidative stress. Our data suggested Syt1 to be a cardiac antioxidant molecule. In addition to oxidative stress, mitochondrial Ca^2+^ overload is another key factor that drives cardiac cell death by triggering mPTP abnormal opening, which contributes to MI, myocardial I/R injury, obesity cardiomyopathy, and cardiac microvascular I/R injury [7, 45, 46]. ROS causes intracellular Ca^2+^ overload by enhancing extracellular Ca^2+^ influx, ultimately leading to Ca^2+^ homeostasis disruption [45]. Syt1 has been identified as a Ca^2+^ sensor [17]. Therefore, we hypothesized that Syt1 regulates mitochondrial Ca^2+^ hemostasis in cardiomyocytes probably via its Ca^2+^ sensor function, which needs to be further confirmed in the future.

Our previous work demonstrated that Parkin, an E3 ubiquitin-protein ligase, could translocate from cytoplasm to mitochondria, where it interacted with CypD and catalyzed CypD ubiquitination, thus suppressed both mPTP opening and cardiomyocyte necrosis under moderate oxidative stress, whereas Parkin was significantly downregulated under severe oxidative stress [15]. In this study, both Syt1 and Parkin were significantly decreased in the mitochondria of cardiomyocytes treated with high concentration of H_2_O_2_. Immunoprecipitation and Immunofluorescence results revealed a strong interaction between Syt1 and Parkin, and the interaction was disrupted by H_2_O_2_ or Syt1 deficiency. We further found that Parkin-catalyzed CypD ubiquitination was attenuated by knockdown of Syt1 and enhanced by overexpression of Syt1. Ubiquitin-activating enzymes (E1), ubiquitin-conjugating enzymes (E2) and ubiquitin-protein ligases (E3) cooperatively catalyze the ubiquitination reaction. Beyond this conventional ubiquitination catalyzation, growing non-enzymatic ubiquitination regulatory factors have been identified, and these factors form ubiquitination or deubiquitination complex with ubiquitinase and substrates [47–49]. In the signaling pathway of cardiac hypertrophy, BAF55 suppresses ubiquitin-protein ligase WWP2-mediated PARP1 ubiquitination [47]. YTH N6-methyladenosine (m6A) RNA binding protein 1 (YTHDF1) translationally upregulated WWP1 and enhanced WWP1-catalyzed NLRP3 ubiquitination [48]. Abraxas brother 1 (ABRO1) acts as a scaffold protein to assemble substrate NLRP3 and ubiquitin-protein ligase BRCC3, and reduces the ubiquitination modification of NLRP3 by inactivating BRCC3 [49]. Our present data showed that Syt1 interacted with Parkin and promoted Parkin-mediated CypD ubiquitination, suggesting that Syt1 is a member of the ubiquitination complex and functions as a ubiquitination regulatory factor. Regarding the underlying mechanisms, we proposed certain hypotheses. One is that Syt1 probably enhances Parkin translation, which could be predicted by the significant upregulation of Parkin protein level by overexpression of Syt1 (shown in Fig. 3a). Syt1 may enhance the enzyme activity of Parkin in catalyzing CypD ubiquitination. Another hypothesis is that Syt1 functions as a scaffold protein to enhance the interaction between Parkin and CypD. In what way does Syt1 affect Parkin-mediated CypD ubiquitination will be studied in detail in the future.

The upstream regulatory mechanisms of Syt1 remain an enigma so far. The present work identified that microRNA (miR)-193b-3p is a novel upstream regulator of Syt1. MicroRNAs, a class of single-stranded and highly-conserved small non-coding RNAs, suppress gene expression at post-transcriptional or translational levels, and is deeply implicated in cardiac development and disorders. Previous studies on the functions of miR-193b-3p were mainly focused on tumor [50, 51], inflammation [52, 53], and metabolism [39, 54]. Recent advances have revealed that miR-193b-3p participates in the regulation of pulmonary hypertension in heart failure [39], cardiac hypertrophy [40] and the apoptotic pathway in myocardial ischemia/reperfusion injury [41]. However, the roles of miR-193b-3p in the cardiomyocyte necrotic death have not been explored. Here we found that miR-193b-3p directly bound to the 3′ untranslated region (UTR) of Syt1 mRNA and downregulated the Syt1 protein level, and by this way miR-193b-3p promoted necrotic cell death and mPTP opening. We also demonstrated that miR-193b-3p regulated cardiomyocyte necrosis, interstitial fibrosis and cardiac function in mice with I/R.

In conclusion, this work demonstrates that Syt1 has a detrimental effect on mPTP opening and cardiomyocyte programmed necrosis, and thus exerts a cardioprotective effect in I/R injury. Mechanistically, Syt1 interacts with Parkin and promotes Parkin-mediated CypD ubiquitination. Syt1 expression is negatively regulated by miR-193b-3p. The study reveals a novel regulatory model of cardiomyocyte necrosis which is composed of miR-193b-3p, Syt1, Parkin, and CypD. These molecules may serve as potential therapeutic targets and modulating the model may become strategies to treat myocardial necrosis-associated heart diseases, including I/R injury, MI and heart failure.

## Supplementary information

Supplementary figures and figure legends

Full and uncropped western blots

## Data Availability

The data underlying this article will be shared on reasonable request to the corresponding author.
