# Correction: Synaptotagmin-1 attenuates myocardial programmed necrosis and ischemia/reperfusion injury through the mitochondrial pathway

**DOI:** 10.1038/s41419-025-07725-7

**Published:** 2025-05-23

**Authors:** Teng Sun, Jialei Li, Shuang Wang, Yu Han, Xiangyu Tao, Min Yuan, Zhijie Jing, Ting Liu, Yuehong Qi, Siqi Liu, Yanlin Feng, Jiasong Chang, Lan Zhou, Lijuan Gao, Jianyun Shi, Ruihong Ning, Jimin Cao

**Affiliations:** 1 Key Laboratory of Cellular Physiology at Shanxi Medical University, Ministry of Education, and the Department of Physiology, School of Basic Medicine, Shanxi Medical University, Taiyuan, China; 2 Laboratory Animal Center, Shanxi Medical University, Taiyuan, China; 3 First Hospital of Shanxi Medical University, Taiyuan, China; 4 The Anesthesiology Department of Shanxi Provincial People’s Hospital, Shanxi Medical University, Taiyuan, China; 5 Department of Cardiology, The Second Hospital of Shanxi Medical University, Taiyuan, China

**Keywords:** Necroptosis, Myocardial infarction, Mitochondria

Correction to: *Cell Death and Disease* 10.1038/s41419-025-07360-2, published online 26 January 2025

After the publication of the article, the authors identified that the image in the third column of Figure 7g was inadvertently misused. The corrected version of Figure 7g is provided below. This correction does not affect the interpretation or the conclusions of the manuscript.
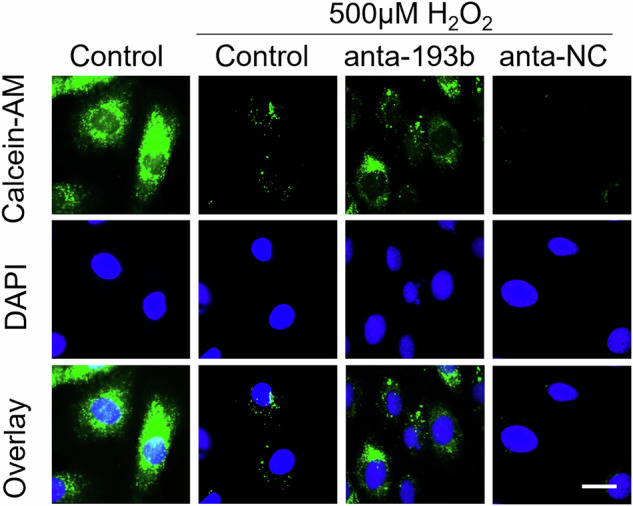


The original article has been corrected.

