# Supplementary material for: Synaptotagmin-1 attenuates myocardial programmed necrosis and ischemia/reperfusion injury through the mitochondrial pathway
Source: Cell Death Dis. 2025 Jan 26;16(1):45. doi: 10.1038/s41419-025-07360-2 (PMC11770119; doi:10.1038/s41419-025-07360-2)
Supplement: Supplementary file 1 — Supplementary figures and figure legends [file 41419_2025_7360_MOESM1_ESM.docx]

**Supplementary Figures and Figure legends**


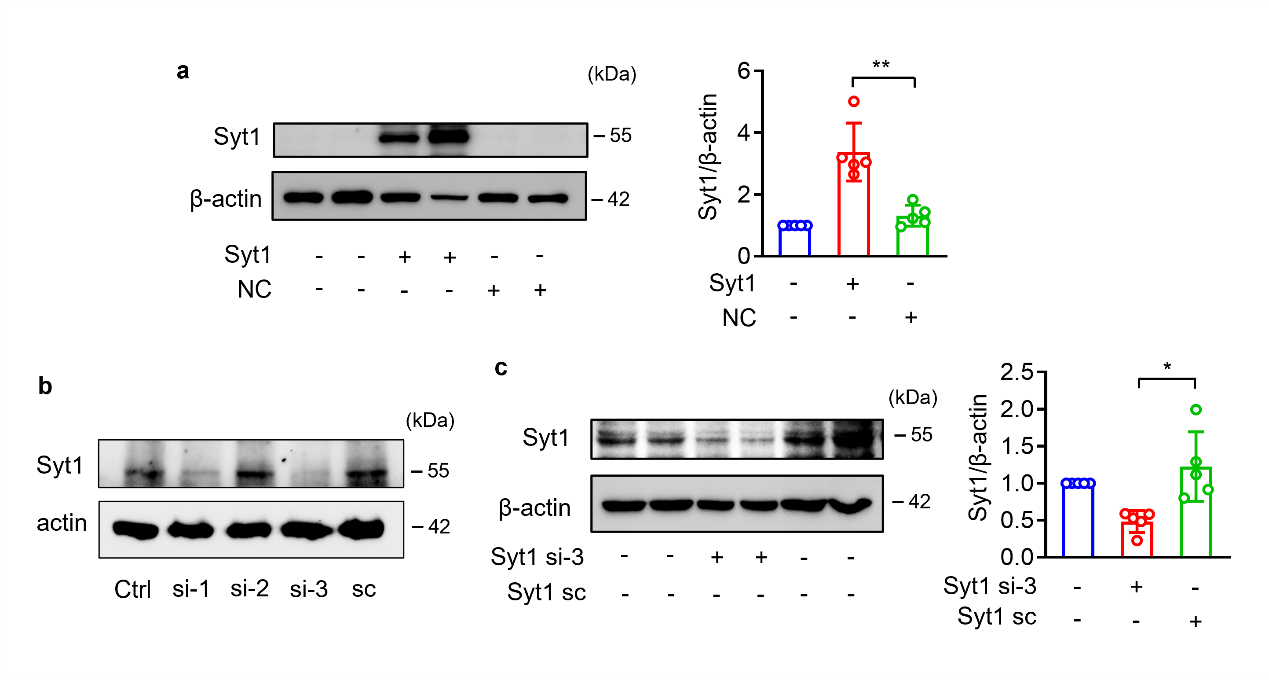


**Supplementary Figure S1. Protein levels of Syt1 detection. a**, Western blotting results showing the protein levels of Syt1 in hearts infected with Syt1 overexpression adenovirus (Syt1) or negative control (NC) for 48h. ** *P* < 0.01. N = 5. **b** and **c**, The protein level of Syt1 detection was performed in cardiomyocytes infected with three types of Syt1 siRNA adenovirus (si-1, si-2, si-3) or scrambled Syt1 adenovirus (Syt1 sc). * *P* < 0.05. N = 5.


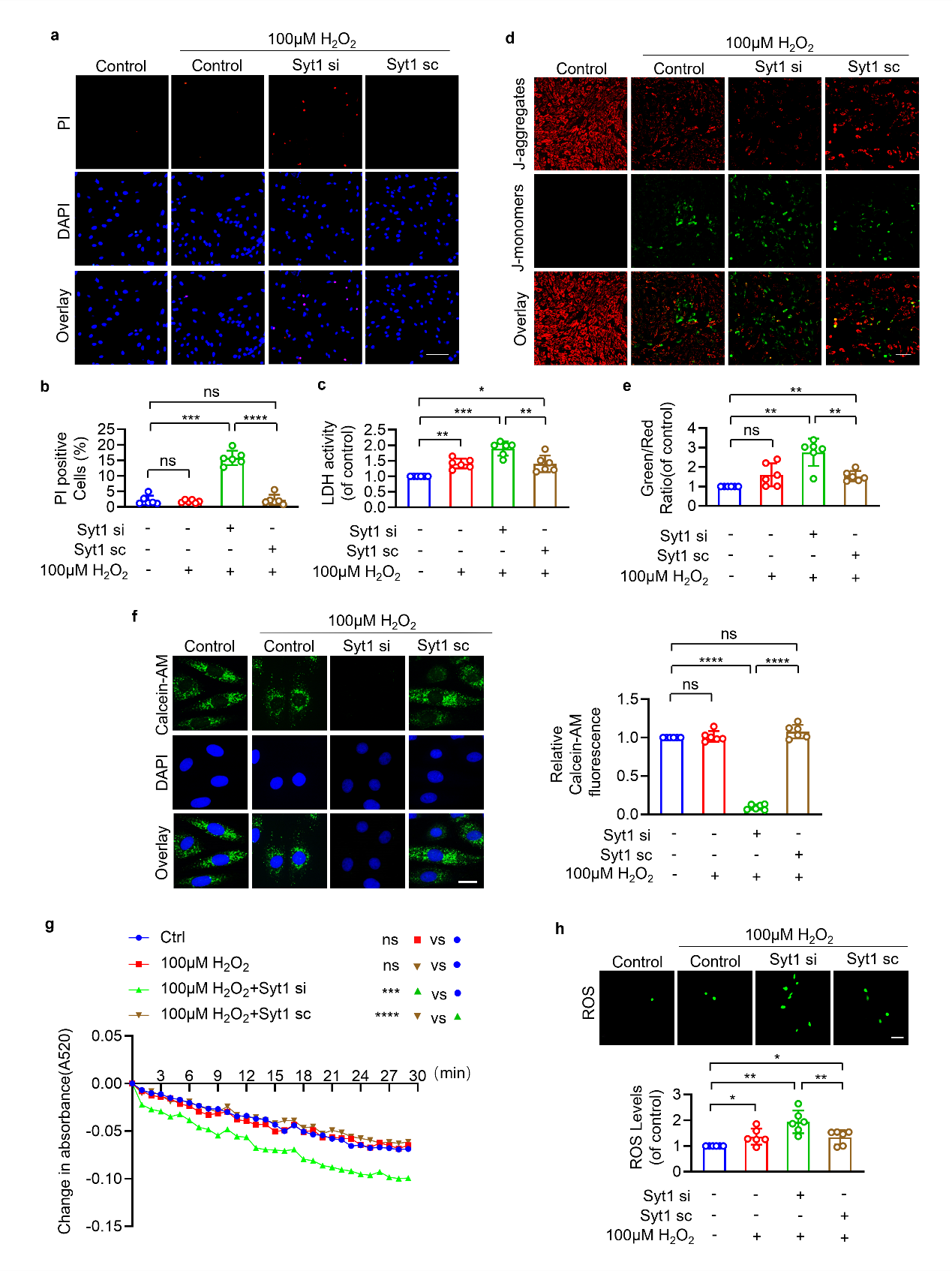


**Supplementary Figure S2. Knockdown of Syt1 sensitized cardiomyocytes to necrosis.** Cardiomyocytes were infected with Syt1 siRNA adenovirus (Syt1 si) or scrambled Syt1 adenovirus (Syt1 sc), and then treated with 100 µM H_2_O_2_. **a** and **b**, PI staining. Bar = 100 µm. *** *P* < 0.001. **** *P* < 0.0001. ns: no statistical significance. N = 6. **c,** LDH activity detection. * *P* < 0.05. ** *P* < 0.01. *** *P* < 0.001. ns: no statistical significance. N = 6. **d** and **e**, mitochondrial membrane potential △Ψm detection was performed using JC-1. The ratio of J-monomers/J-aggregates was calculated. Bar=100 µm. ** *P*<0.01. ns: no statistical significance. N = 6. **f** and **g**, mPTP opening evaluation. (f) The calcein-AM fluorescence intensity detection. Bar = 50 µm. **** *P* < 0.0001. N = 6. (g) Mitochondrial swelling was analyzed by monitoring the absorbance at 520 nm every 1 minute for a total of 30 minutes. *** *P* < 0.001. **** *P* < 0.0001. N = 6. **h**, ROS was detected by fluorescent probe DCFH-DA. Bar = 100 µm. * *P* < 0.05. ** *P* < 0.01. N = 6. These data are expressed as the mean ± SD.


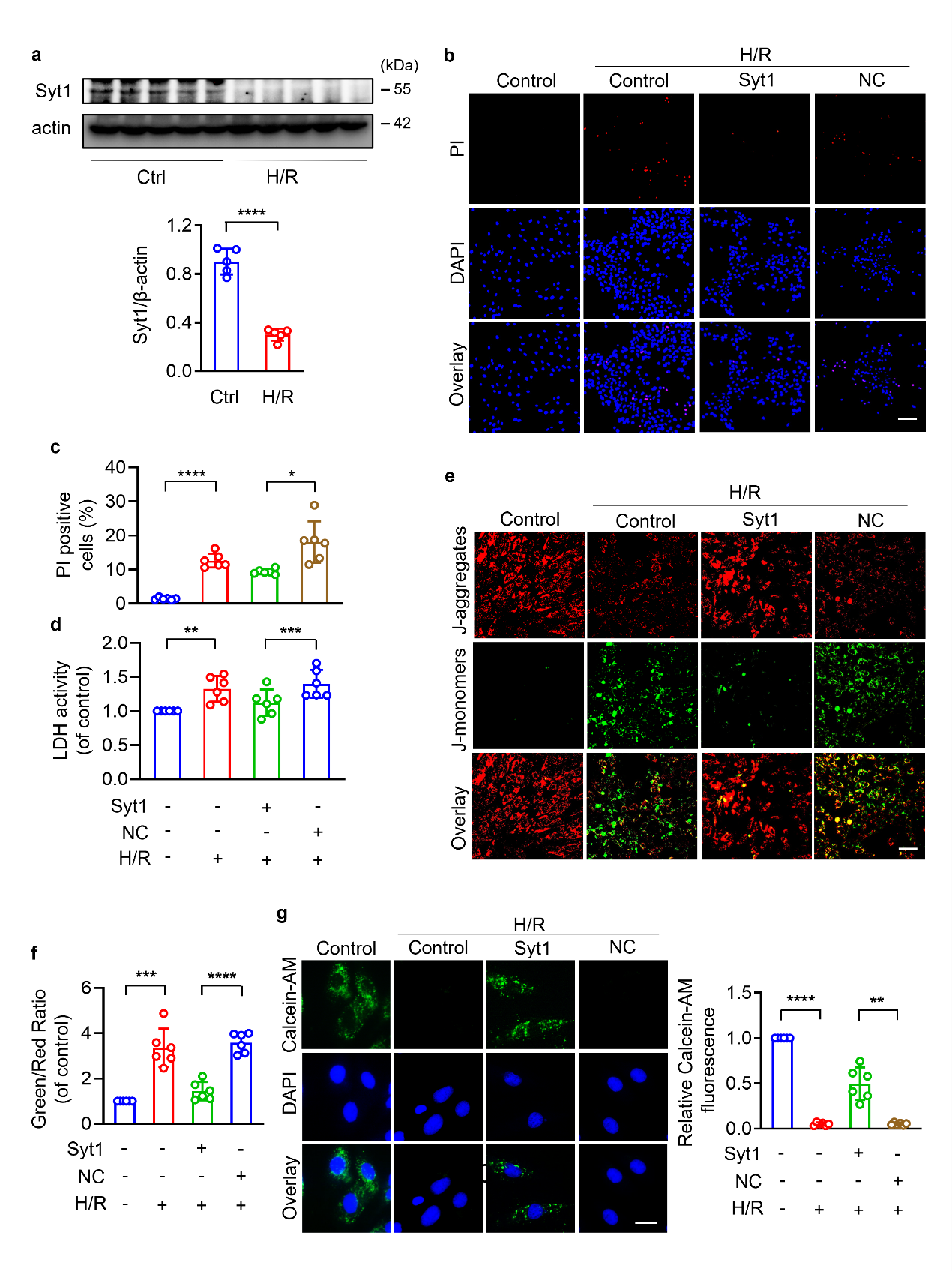


**Supplementary Figure S3. Syt1 suppressed H/R-induced cardiomyocyte necrosis and mPTP opening. a**, Western blotting showing the protein levels of Syt1 in cardiomyocytes treated with hypoxia/reoxygenation (H/R). **** *P* < 0.0001. N = 5. **b−d**, Necrotic cell death detection. Cardiomyocytes were infected with Syt1 overexpression adenovirus (Syt1) or negative control (NC), and then treated with H/R. PI staining. Bar = 100 µm. * *P* < 0.05. **** *P* < 0.0001. N = 6. LDH activity was analyzed. ** *P* < 0.01. *** *P* < 0.001. N = 6. **e** and **f**, Analysis of mitochondrial membrane potential by JC-1. The ratio of J-monomers/J-aggregates was calculated. Bar = 100 µm. *** *P* < 0.001. **** *P* < 0.0001. N = 6. **g**, mPTP opening analysis. The calcein-AM fluorescence intensity detection. Bar = 50 µm. ** *P* < 0.01. **** *P* < 0.0001. N = 6. These data are expressed as the mean ± SD.


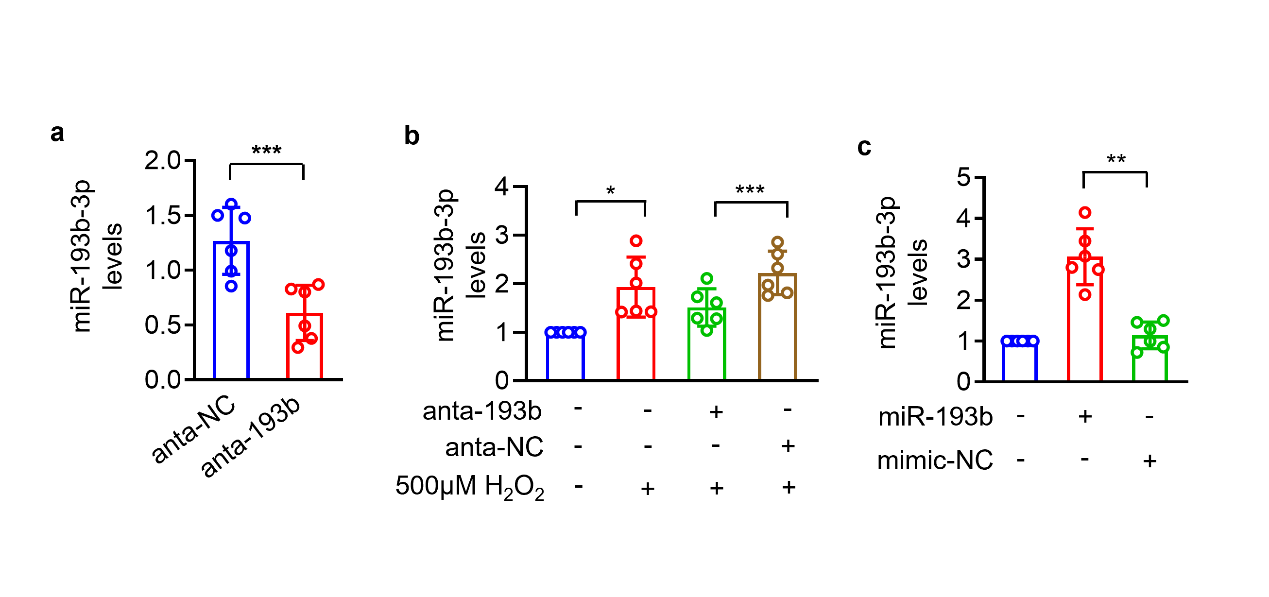


**Supplementary Figure S4. The level of miR-193b-3p detection. a**, The level of miR-193b-3p in hearts injected with miR-193b-3p antagomir adenovirus (anta-193b) or negative control (anta-NC). *** *P* < 0.001. N = 6. **b**, The level of miR-193b-3p was detected in cardiomyocytes infected with miR-193b-3p antagomir adenovirus (anta-193b) or negative control (anta-NC), followed by exposed to 500 µM H_2_O_2_. * *P* < 0.05. *** *P* < 0.001. N = 6. **c**, The levels of miR-193b-3p in cardiomyocytes transfected with miR-193b-3p mimic (miR-193b) or negative control (mimic-NC). ** *P* < 0.01. N = 6. These data are expressed as the mean ± SD.


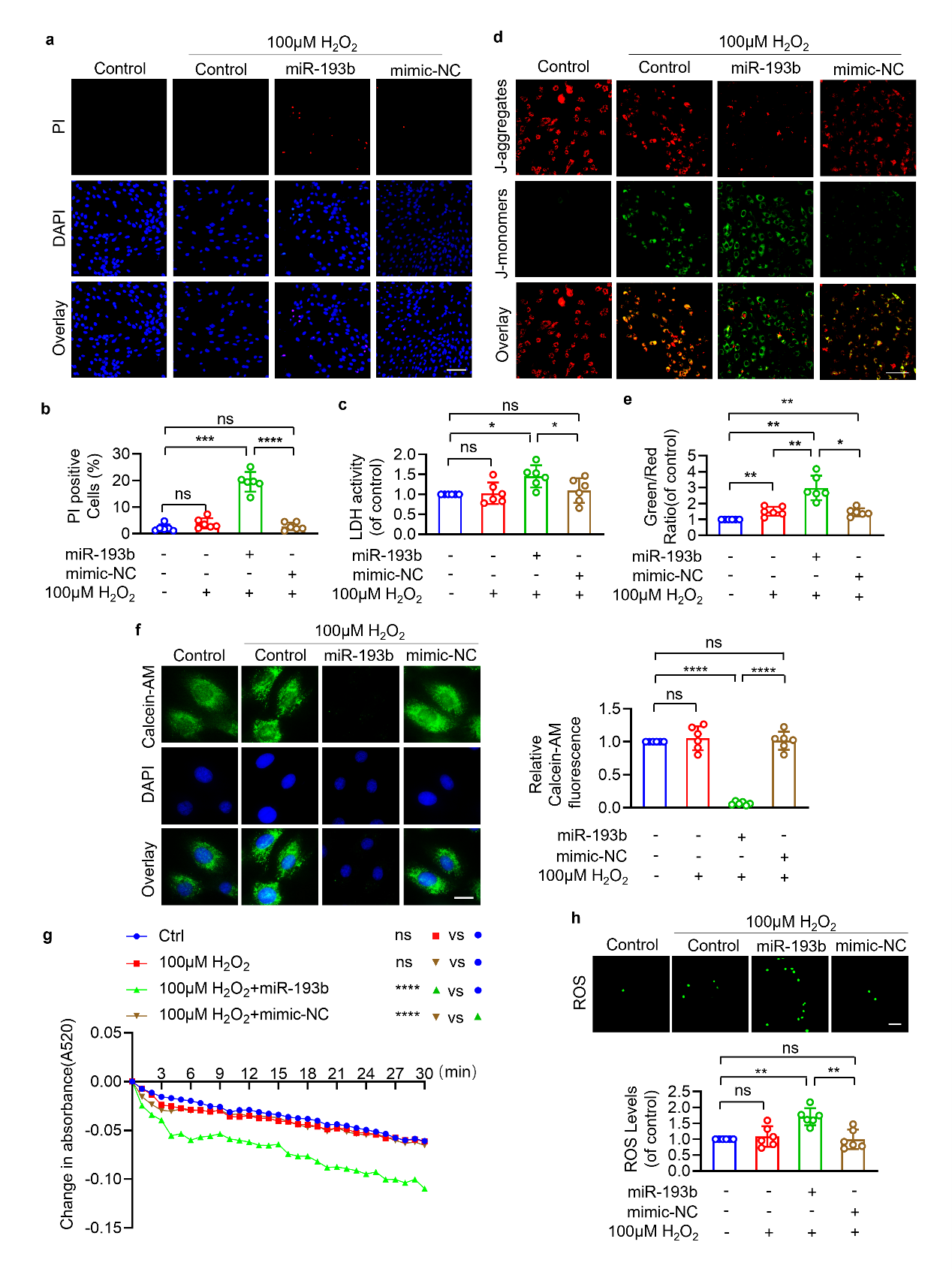


**Supplementary Figure S5. Overexpression of miR-193b-3p increased the susceptibility of cardiomyocytes to necrosis.** Cardiomyocytes were transfected with miR-193b-3p mimic (miR-193b) or negative control (mimic-NC), and then exposed to 100µM H_2_O_2_. **a** and **b**, PI staining. Bar = 50 µm. *** *P* < 0.001. **** *P* < 0.0001. ns: no statistical significance. N = 6. **c,** LDH activity detection. * *P* < 0.05. ns: no statistical significance. N = 6. **d** and **e**, Mitochondrial membrane potential △Ψm was detected using JC-1. The ratio of J-monomers/J-aggregates was calculated. Bar = 100 µm. * *P* < 0.05. ** *P* < 0.01. ns: no statistical significance. N = 6. These data are expressed as the mean ± SD. **f** and **g**, mPTP opening analysis. (f) The calcein-AM fluorescence intensity detection. Bar = 50 µm. **** *P* < 0.0001. N = 6. (g) Mitochondrial swelling was analyzed by monitoring the absorbance at 520 nm every 1 minute for a total of 30 minutes. **** *P* < 0.0001. N = 6. **h**, ROS was detected by fluorescent probe DCFH-DA. Bar = 100 µm. ** *P* < 0.01. N = 6. ns: no statistical significance. These data are expressed as the mean ± SD.


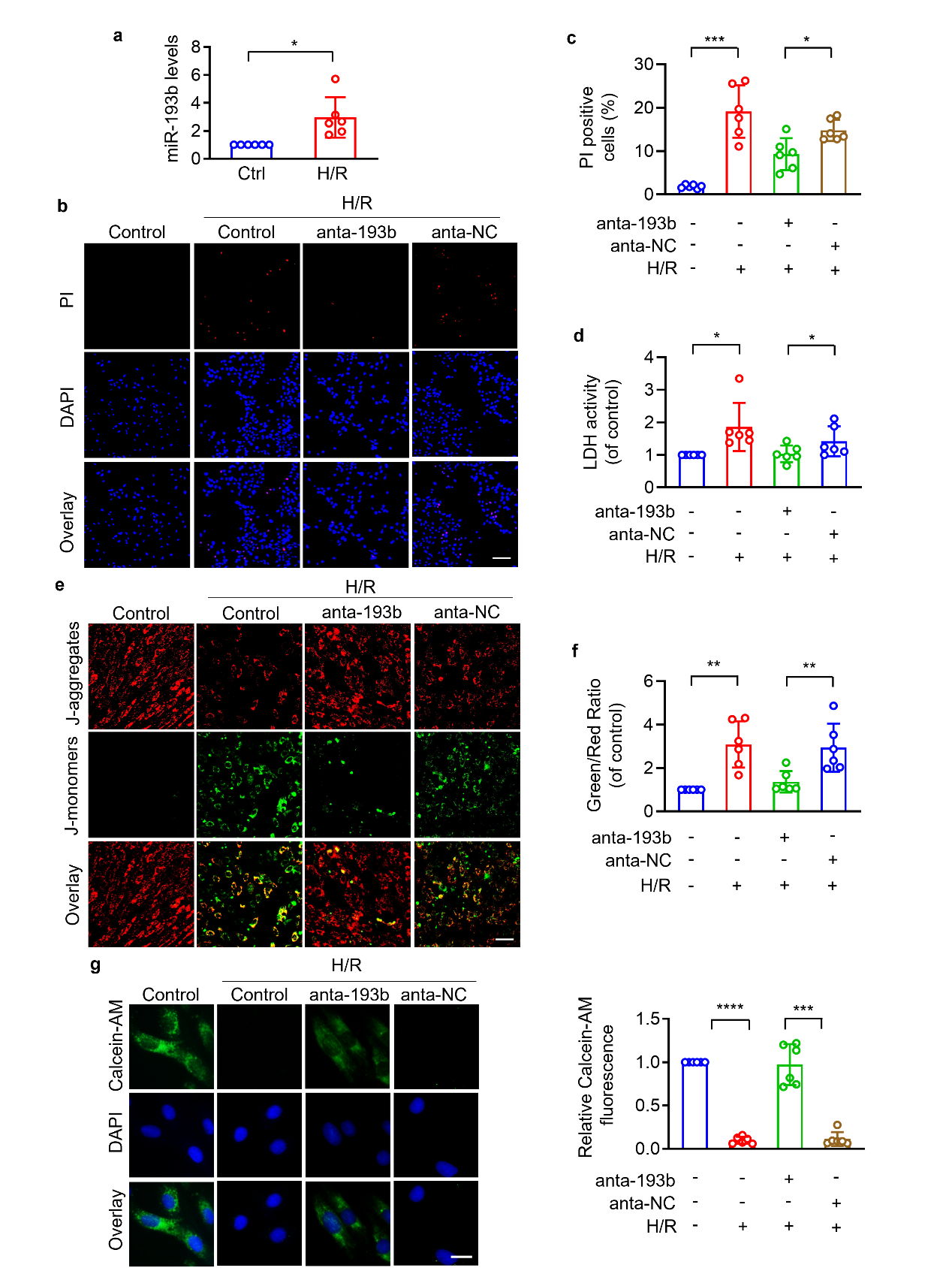


**Supplementary Figure S6. miR-193b-3p regulated** **H/R-induced cardiomyocyte necrosis and mPTP opening. a**, The levels of miR-193b-3p were detected in cardiomyocytes treated with H/R by qRT-PCR. * *P* < 0.05. N = 6. **b−d**, Necrotic cell death analysis. Cardiomyocytes were infected with miR-193b-3p antagomir adenovirus (anta-193b) or negative control (anta-NC), and then treated with H/R. PI staining was performed. Bar = 100 µm. * *P* < 0.05. *** *P* < 0.001. N = 6. LDH activity was analyzed. * *P* < 0.05. N = 6. **e** and **f**, Mitochondrial membrane potential detection by JC-1 staining. The ratio of J-monomers/J-aggregates was calculated. Bar = 100 µm. ** *P* < 0.01. N = 6. **g**, mPTP opening evaluation. The calcein-AM fluorescence intensity detection. Bar = 50 µm. *** *P* < 0.001. **** *P* < 0.0001. N = 6. These data are expressed as the mean ± SD.
