# Supplementary material for: Synaptotagmin-1 attenuates myocardial programmed necrosis and ischemia/reperfusion injury through the mitochondrial pathway
Source: Cell Death Dis. 2025 Jan 26;16(1):45. doi: 10.1038/s41419-025-07360-2 (PMC11770119; doi:10.1038/s41419-025-07360-2)

Figure 1-a

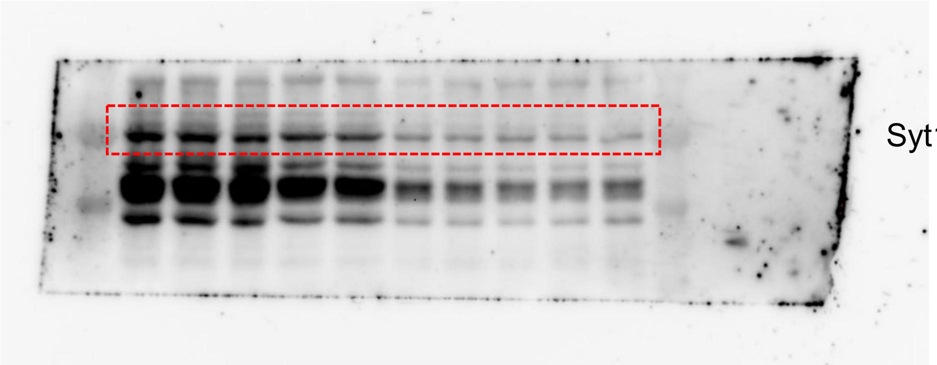

Syt1

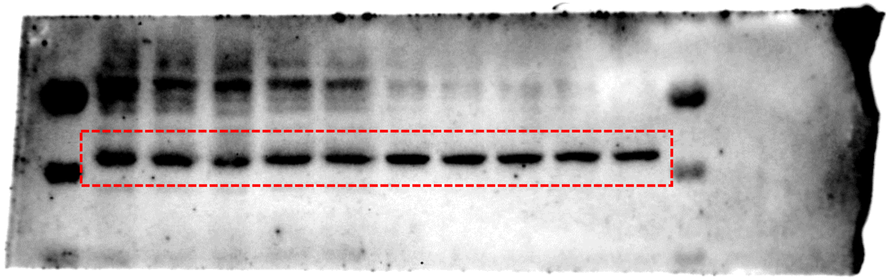

β-actin

Figure 2-a

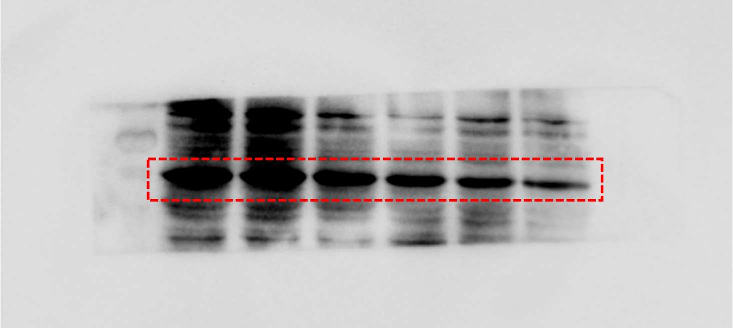

Syt1

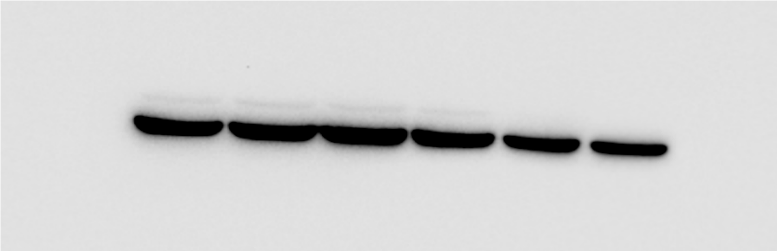

β-actin

Figure 2-b

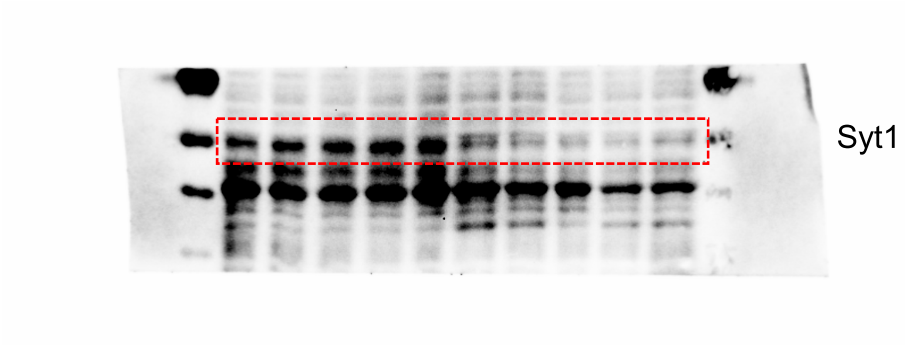

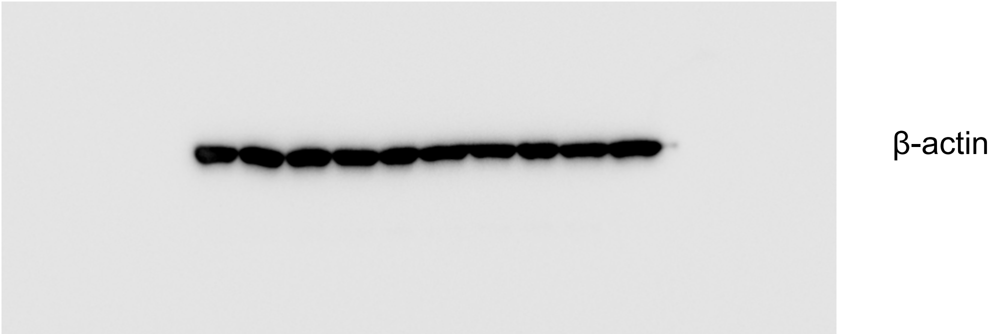

Figure 3-a

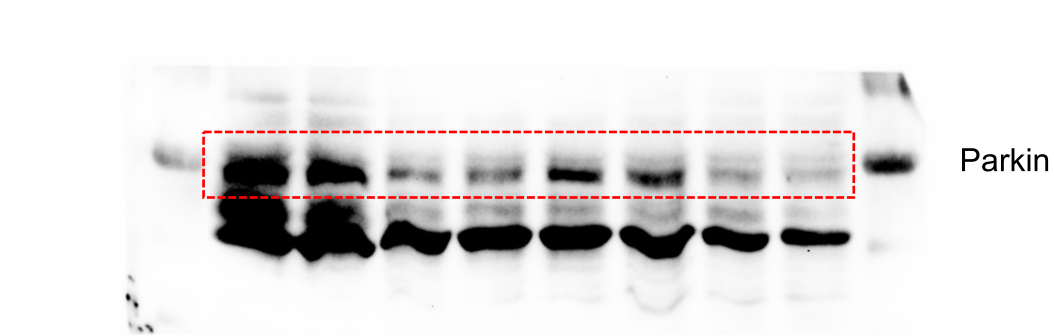

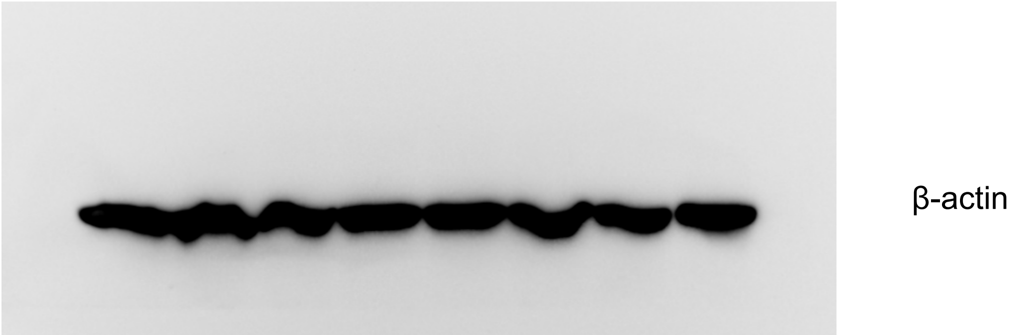

Figure 3-c

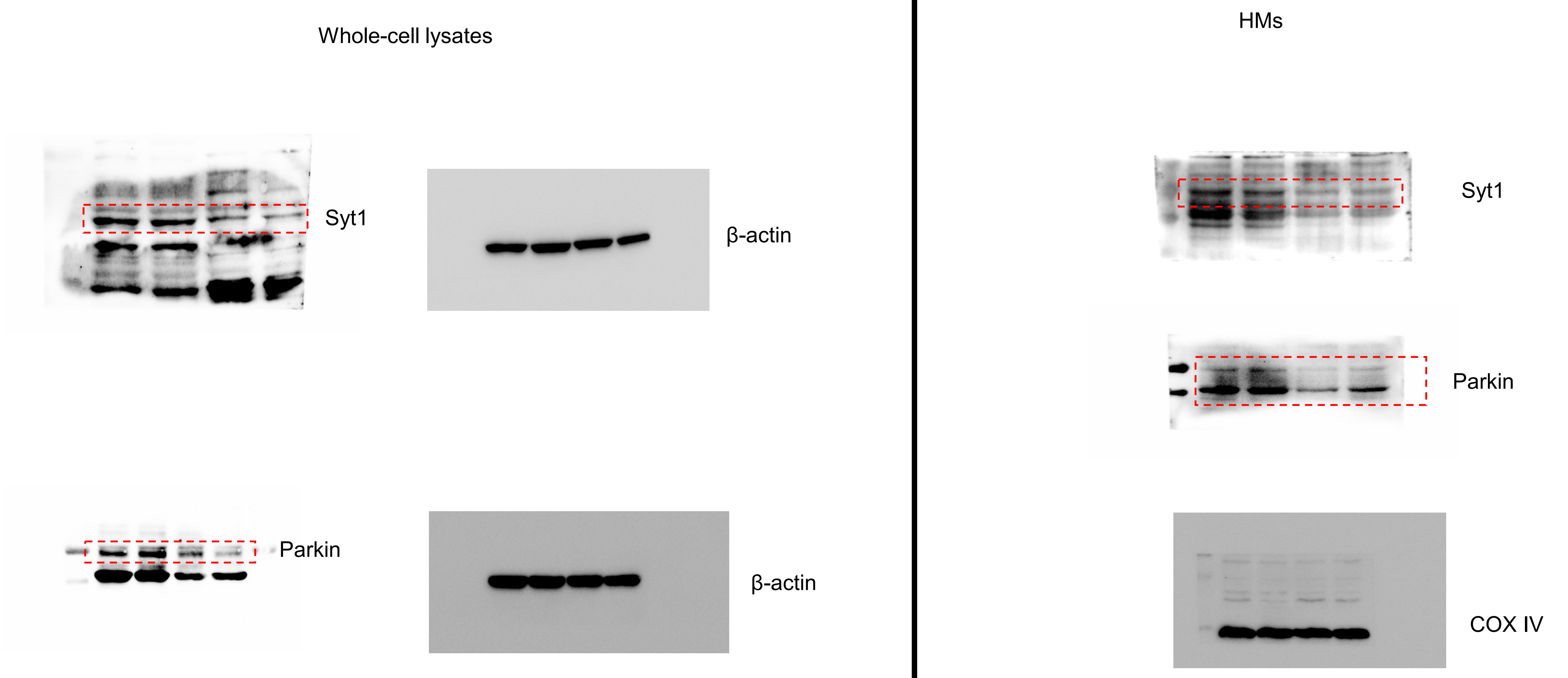

Whole-cell lysates
Syt1
β-actin
Parkin
β-actin
HMs
Syt1
Parkin
COX IV

Figure 3-d

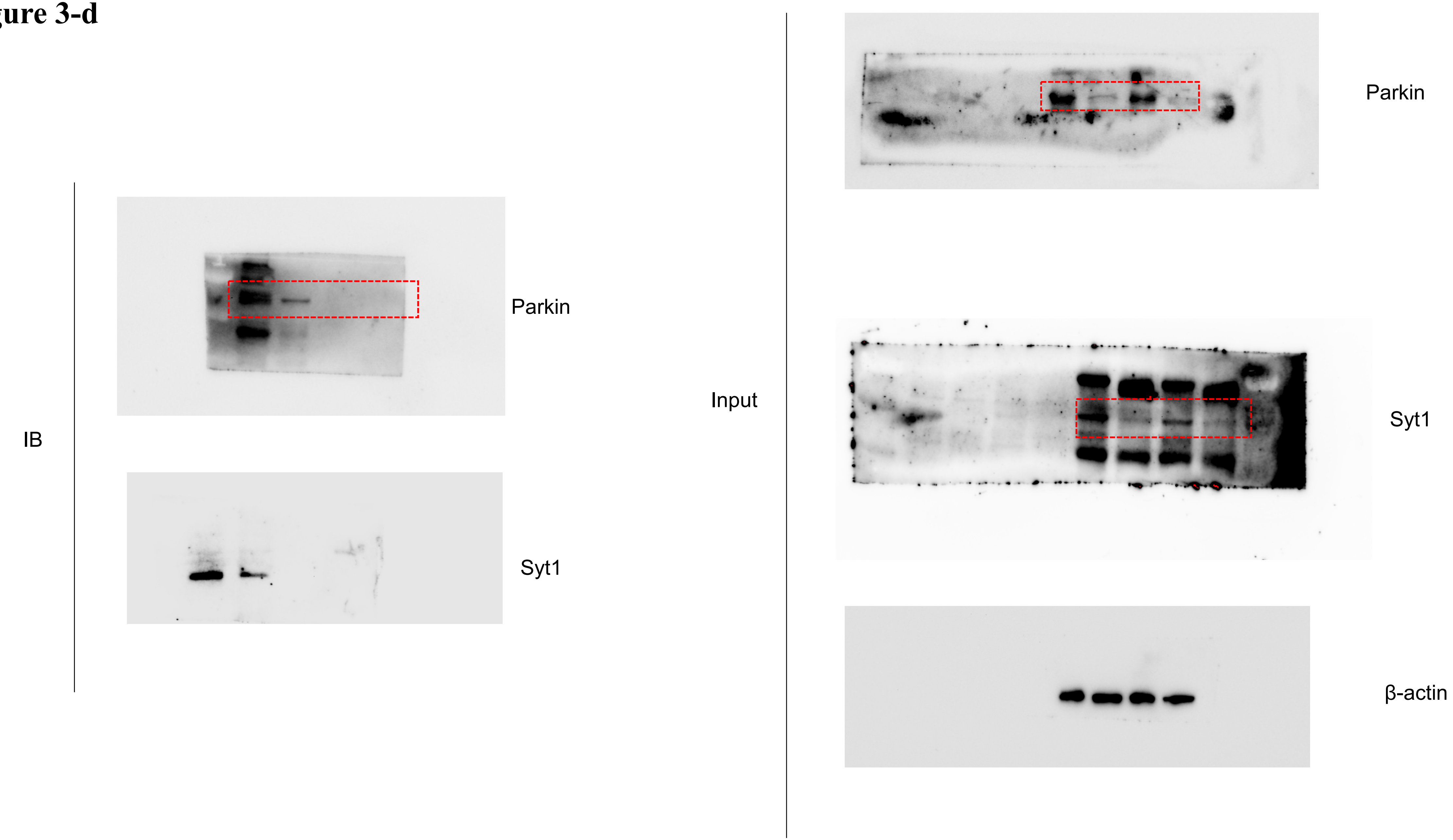

Figure 3-e

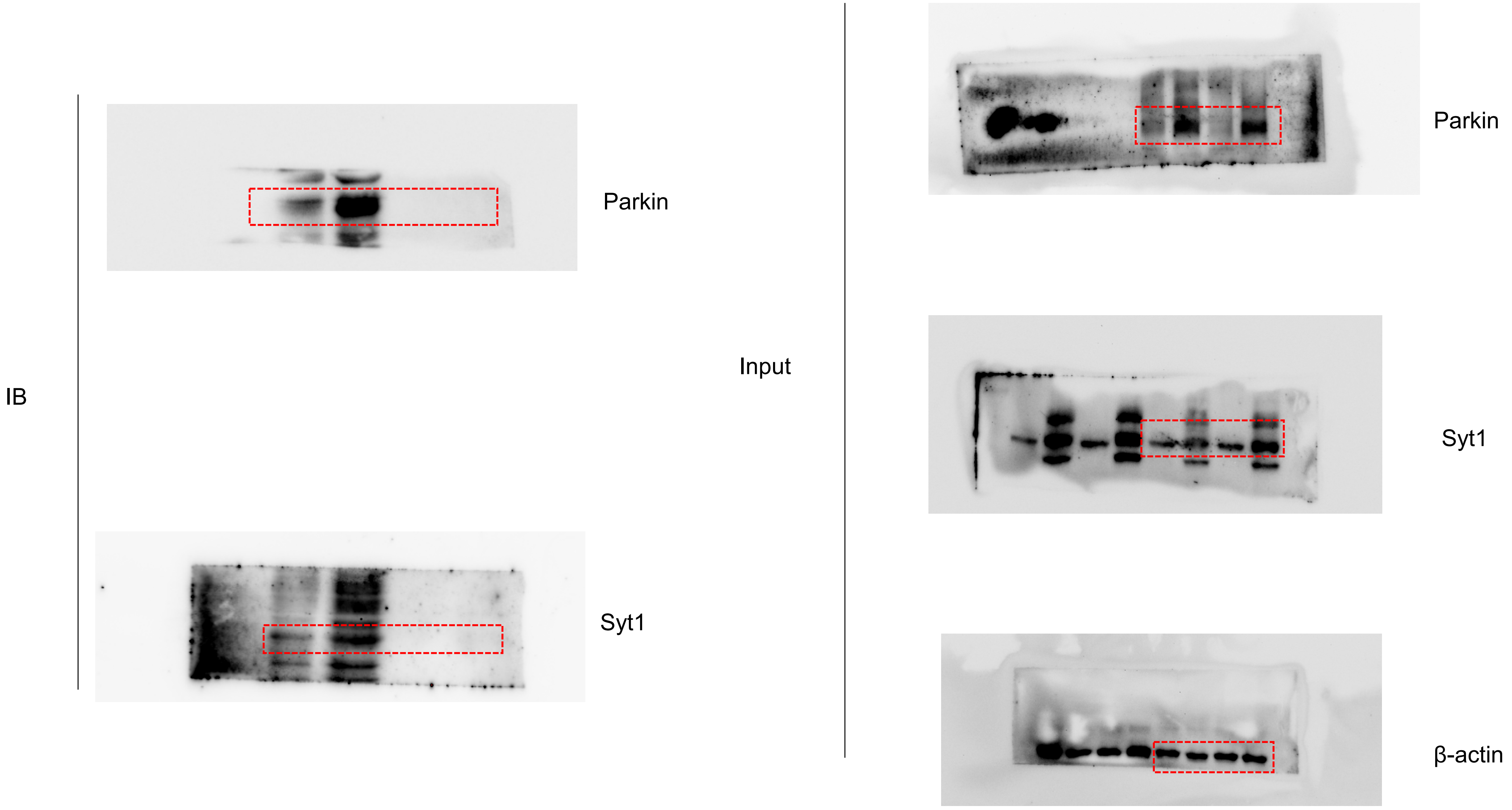

Figure 3-f

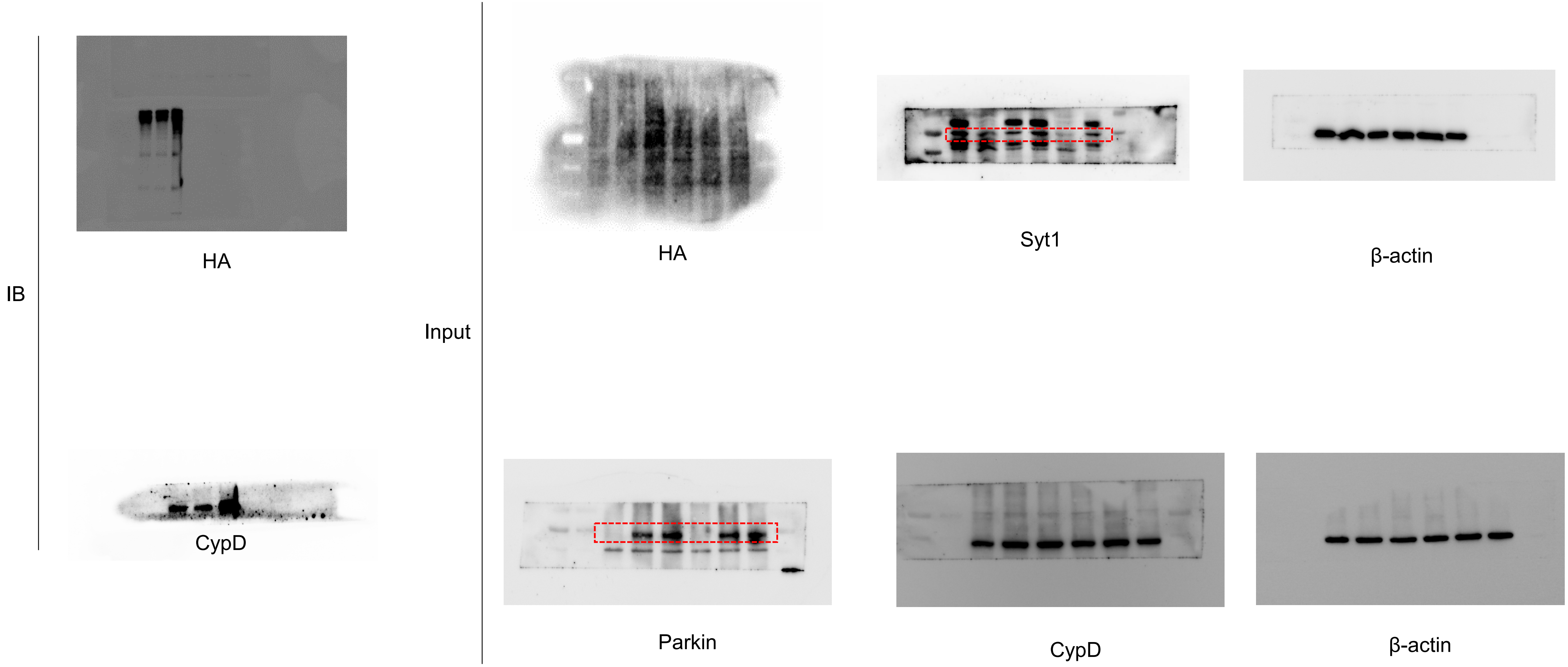

Figure 3-g

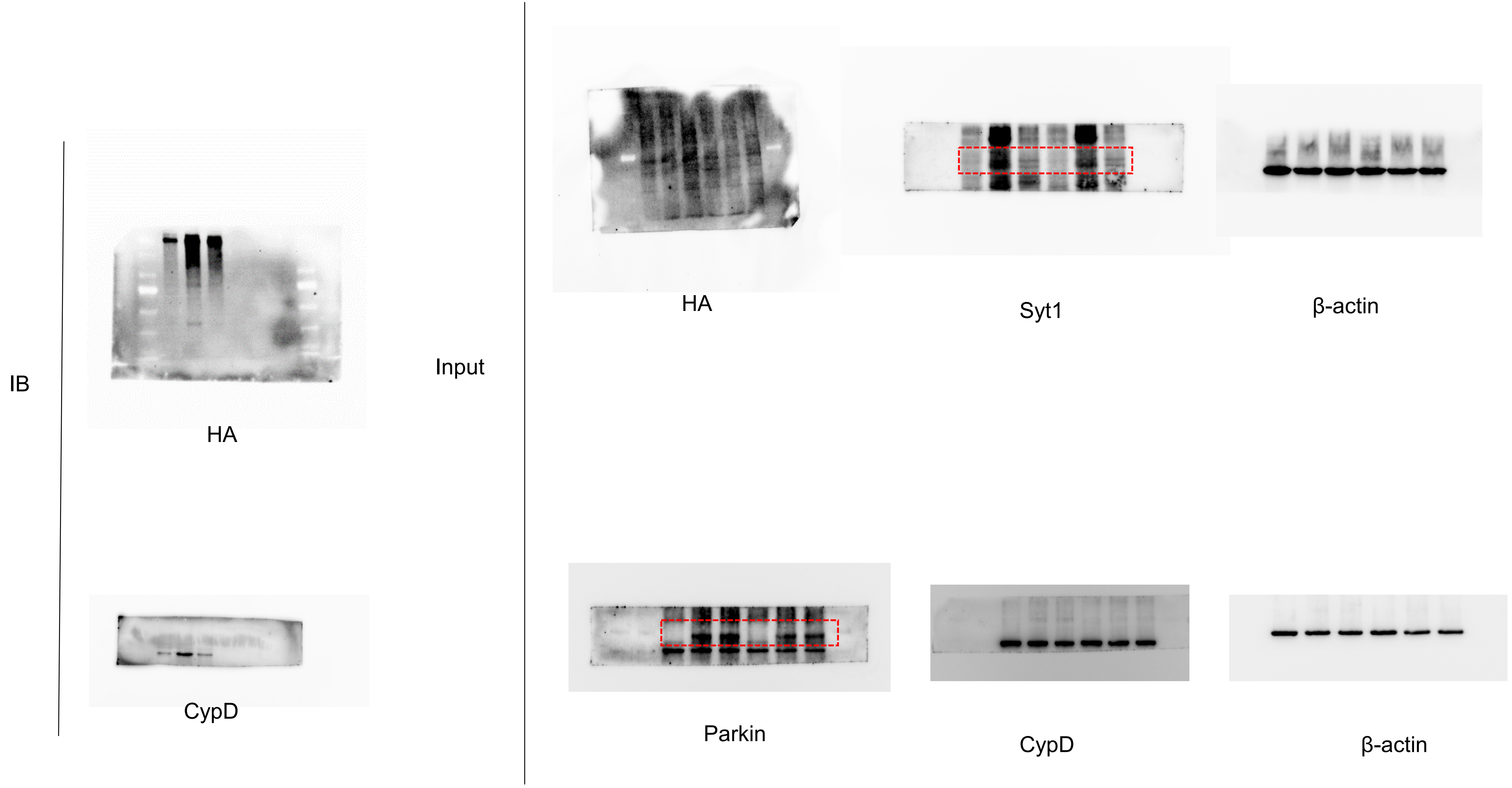

Figure 5-b

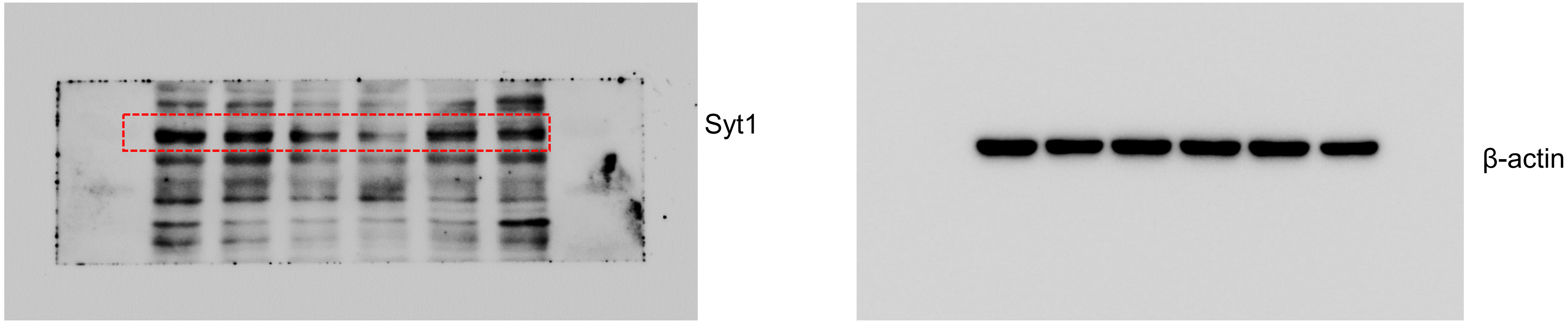

Figure 5-c

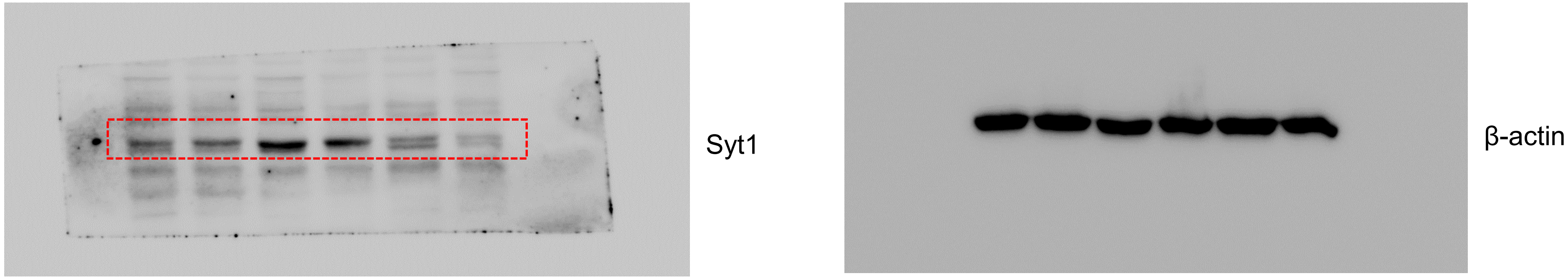

Supplementary Figure S1-a

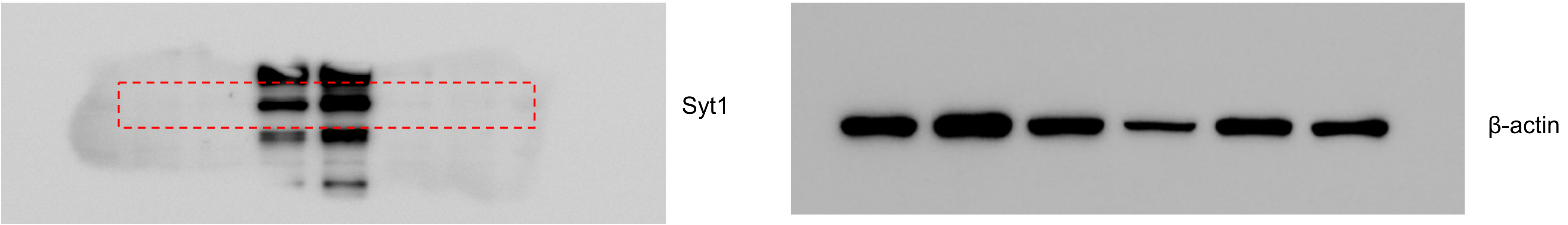

Supplementary Figure S1-b

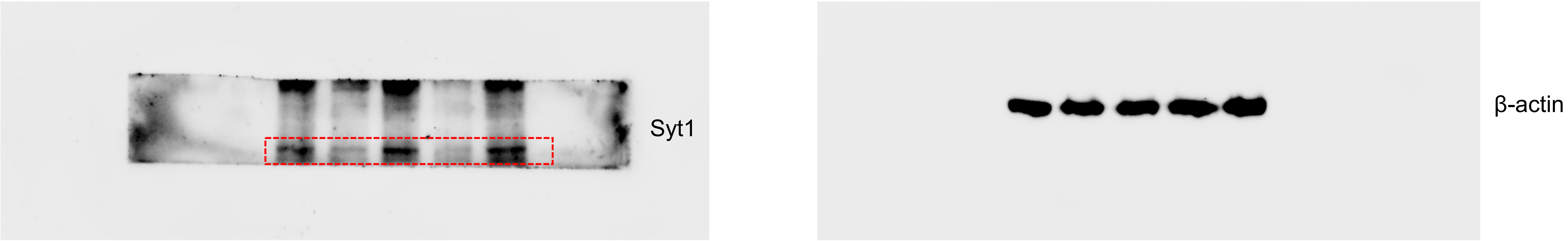

Supplementary Figure S1-c

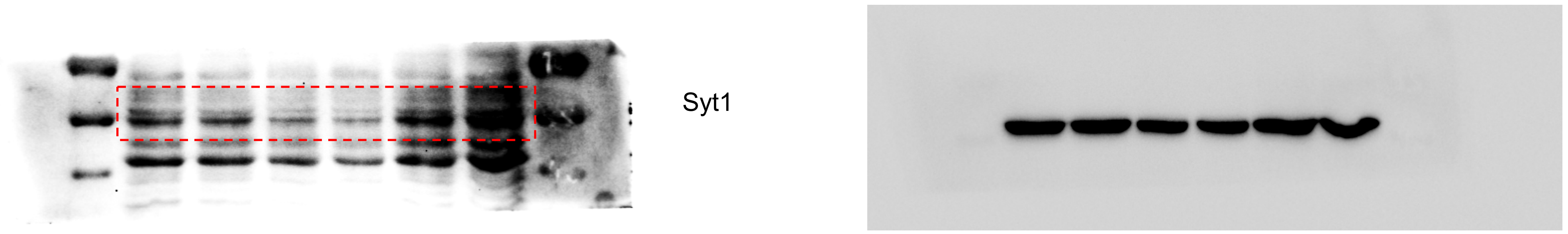

Supplementary Figure S3-a

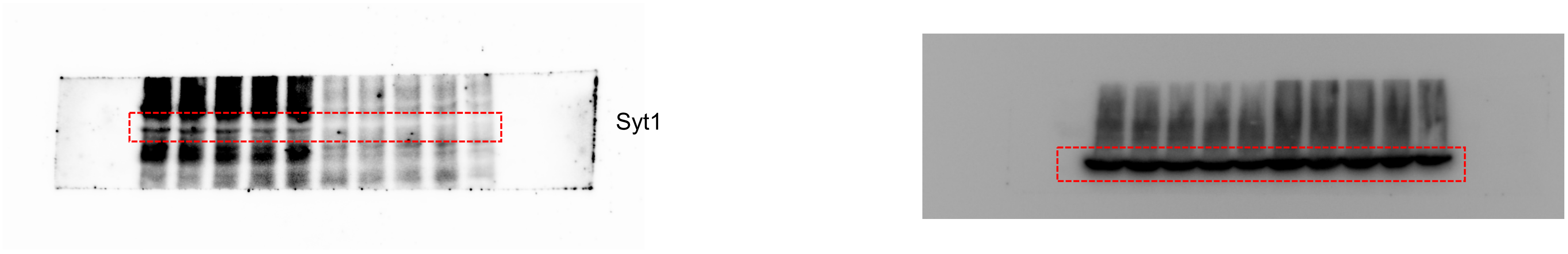

Supplement: Supplementary file 2 — Full and uncropped western blots [file 41419_2025_7360_MOESM2_ESM.pdf]
